# Atypical IκB Bcl3 enhances the generation of the NF-κB p52 homodimer

**DOI:** 10.3389/fcell.2022.930619

**Published:** 2022-08-05

**Authors:** Wenfei Pan, Limei Deng, Haitao Wang, Vivien Ya-Fan Wang

**Affiliations:** ^1^ Faculty of Health Sciences, University of Macau, Avenida da Universidade, Macau SAR, China; ^2^ Thoracic Surgery Branch, Clinical Research, Center for Cancer Research, National Cancer Institute, National Institute of Health, Bethesda, MD, United States; ^3^ Cancer Centre, Faculty of Health Sciences, University of Macau, Avenida da Universidade, Macau SAR, China; ^4^ MoE Frontiers Science Center for Precision Oncology, University of Macau, Avenida da Universidade, Macau SAR, China

**Keywords:** NF-κB—nuclear factor-kappa B, dimerization, Bcl3, NF-κB:IκB complex, gene expression

## Abstract

The NF-κB family of dimeric transcription factors regulate diverse biological functions. Their cellular expression profiles differ, which lead to different concentrations in different cell/tissue types. Although the activation mechanisms of different NF-κB dimers have been widely investigated, there is limited information on specific NF-κB dimers’ formation. The NF-κB p52:p52 homodimer regulates an important subset of target genes in cancer cells; however, the molecular mechanism of the generation of this specific homodimer remains unclear. Our study has revealed that the atypical IκB protein, Bcl3, plays an essential role in enhancing the p52:p52 homodimer population which is a unique mechanism to p52 within the NF-κB family. p52 was shown to heterodimerize with four other NF-κB subunits (RelA, RelB, cRel, and p50); all heterodimers, except p52:p50, are significantly more stable than the p52:p52 homodimer. Bcl3 is able to compete with all other NF-κB subunits in cells for efficient p52:p52 homodimer formation which consequently leads to the upregulation of target genes that are involved in cell proliferation, migration, and inflammation, which explain why aberrant activation of Bcl3 and p52 leads to cancer.

## Introduction

Bcl3, B-cell leukemia/lymphoma 3, was identified as an oncoprotein in a subgroup of B cell lymphocytic leukemias with t (14; 19) chromosomal translocation (Ohno et al., 1993). In many cancers, Bcl3 levels are 3–4-fold higher than non-cancerous tissues or cells (Nishikori et al., 2003; Mathas et al., 2005). Structurally, Bcl3 belongs to the inhibitor of the NF-κB (IκB) protein family. There are several members of this family including IκBζ and IκBNS that perform similar functions as Bcl3. They form stable complexes with two dimers of the NF-κB transcription factor family; p52:p52 and p50:p50 homodimers. The p50 and p52 subunits lack a transcriptional activation domain (TAD) and thus are thought to be repressors of transcription. Bcl3, IκBζ, and IκBNS act as transcriptional coregulators by forming ternary complexes with p50:p50 and/or p52:p52 homodimers and DNA response elements (Fiorini et al., 2002; Trinh et al., 2008).

RelA/p65, RelB, and cRel are the other three members of the NF-κB family. They are distinct from p50 and p52 by having a TAD. All five subunits share highly homologous sequences near their N-termini DNA binding domain referred to as the rel homology region (RHR) (Supplementary Figure S1A). The RHR is folded into three structural regions: the N-terminal domain (NTD), dimerization domain (DD), and nuclear localization signal (NLS). The DD alone mediates the association of NF-κB subunits to form combinatorial dimers; the NTD and DD together perform the sequence-specific DNA binding; and the DD and NLS regions are the primary binding sites for IκB proteins. Fifteen NF-κB homo- and heterodimers could be formed through pairwise combination of the five NF-κB subunits; however, the function of every potential dimer is not known. Some of the dimers do not exist due to differential kinetics of expression, or less abundant because of their differential affinity of formation. In addition, the cell type–specific expression also determines why some of the dimers are not observed in specific cells.

Despite the strong structural similarity and DNA binding mechanism (Ghosh et al., 1995; Cramer et al., 1997; Chen et al., 1998a; Chen et al., 1998b; Moorthy et al., 2007; Fusco et al., 2009), NF-κB dimers activate overlapping but different subsets of target genes. Dimers generated from the canonical NF-κB signaling pathway (p50:RelA, RelA:RelA, p50:cRel, and cRel:cRel) in response to inflammatory signals or pathogen-derived substances are mainly responsible for the activation of inflammatory genes, while another group of dimers, predominantly the p52:RelB heterodimer, generated from the non-canonical NF-κB signaling pathway, are involved in the activation of immune developmental genes (Shih et al., 2011). Since the processing of NF-κB2/p100 into p52 is stringently regulated, requiring specific cell signaling events and RelB is thought to be an obligate partner of the precursor p100 protein, formation of the p52:p52 homodimer has remained an unanswered question. It is, however, undeniable that this homodimer is an essential NF-κB regulating the expressions of several target genes.

It has been known for nearly 30 years that the atypical IκB protein, Bcl3, is a partner of both p52:p52 and p50:p50 homodimers, and the trimeric complexes (p52:p52:Bcl3 and p50:p50:Bcl3) mediate gene transcription (Bours et al., 1993; Fujita et al., 1993; Inoue et al., 1993). Several publications since then have highlighted the importance of this complex in both normal and pathogenic cell physiology. Bcl3’s role in immune and inflammatory pathologies is well known. Bcl3 is linked to promoting tumor cell proliferation, survival, invasion, and metastasis and thus is used as a diagnostic marker for various blood and solid tumors. It is reported that in glioblastoma, the p52:p52 homodimer, together with Bcl3, activated genes induce more malignant phenotype cell differentiation (Wu et al., 2018). In breast cancer, p52 and Bcl3 are found to promote cell proliferation by upregulating cyclin D1 gene expression (Cogswell et al., 2000; Westerheide et al., 2001). p52 and Bcl3 are also reported to transactivate apoptosis-related genes (Viatour et al., 2003).

Since the p50:p50 homodimer is formed constitutively, the association between p50 and Bcl3 is relatively easy to comprehend. However, the mechanism of the p52:p52:Bcl3 complex formation *in vivo* is unknown. Also, it is not clear if Bcl3 prefers one of these two homodimers for association. Moreover, it has not been tested whether some of the unobserved dimers, such as p50:p52 or p52:cRel, can form in cells. The present study focuses on the mechanism of specific association between the p52:p52 homodimer and Bcl3 in cells. We addressed the mechanism of NF-κB dimer formation in the competing cellular environment using a multicolor bimolecular fluorescence complementation (BiFC) system. Our results revealed that very little p52:p52 homodimer is generated in the presence of RelB; however, the presence of Bcl3 enhances the cellular population of this homodimer. Bcl3 is also able to compete with all the other p52 containing dimers (p52:RelA, p52:cRel, and p52:p50) in cells for efficient p52:p52 homodimer formation which consequently leads to the upregulation of genes targeted by the p52:p52:Bcl3 complex. We also found that all p52 heterodimers are more stable than the p52:p52 homodimers, except the p50:p52 heterodimer.

## Materials and methods

### Antibodies and reagents

RelB (BB-AB0076C), p50 (BB-AB0080C), p52 (AB0085C), H3 (BB-AB0055C), tubulin (BB-AB0118C), GAPDH (BB-AB0060) antibodies and Protein A PLUS agarose bead (BB-PA001PE) were purchased from BioBharati LifeScience (BBL), Kolkata, India. NF-κB p65/RelA (D14E12) XP antibody and HA (3,724) antibody were purchased from Cell Signaling Technology. Bcl3 (23959-1-AP-100UL) antibody was purchased from Proteintech. Flag M2 (F3165) antibody was purchased from Sigma-Aldrich. Goat anti-mouse IgG-HRP (sc-2302) and goat anti-rabbit IgG-HRP (sc-2030) were purchased from Santa Cruz. The dual-luciferase reporter assay system (E1910) was purchased from Promega. The SuperSignal West Pico Chemiluminescent Substrate kit (34,580) was purchased from ThermoFisher.

### Transient protein expression in mammalian cell culture

HEK 293T and HeLa cells were cultured in a DMEM (Gibco) supplemented with 10% fetal bovine serum (FBS; Gibco), 2 mM glutamine, and 100 U/ml penicillin/streptomycin (Corning) at 37°C with 5% CO_2_. Cells were seeded to the 6-well or 12-well plate the day before transfection. DNA (μg) and 1 mg/ml polyethylenimine (PEI; μL) were mixed at a ratio of 1:2 and added to the cells. Cells were prepared for subsequent experiments 48 h after transfection.

### Multicolor bimolecular fluorescence complementation assay

The sequences encoding amino acid residues 1–172 of Venus or 155–238 of Cerulean were fused to the C-terminus of p52 (1–341) or p50 (1–363) using a linker sequence encoding (GGGGS)_2_, termed as p52-VN and p52-CC, p50-VN and p50-CC, respectively; 1–172 of Cerulean was fused to the C-terminus of RelB (1–400), RelA (1–325), or cRel (1–295) using the same linker, termed as RelB-CN, RelA-CN, or cRel-CN, respectively. Sterilized coverslips were placed into wells of a 6-well plate. HEK 293T cells were seeded to the plate with coverslips. Different combinations of NF-κB multicolor BiFC constructions were co-transfected into HEK 293T cells with or without Bcl3. The cell culture medium was removed 48 h after transfection; cells were washed with ice-cold PBS and fixed by ice-cold pure methanol at −20% for 15 min, followed by washing with ice-cold PBS. The coverslip was removed from the well and mounted to a slide by ProLong Diamond Antifade Mountant (Invitrogen; P36965). The fluoresce signal was imaged by Carl Zeiss Confocal LSM710. Venus and Cerulean signals were captured by the excitation laser at 514 and 405 nm, respectively. Images were acquired with 40 × 1.3 N.A. oil Plan-Neofluar objective. The fluorescent intensity was quantified by Image-ProPlus (IPP) software. Briefly, the fluorescent images were converted to grayscale 8 and inverted to contrast; IOD (integrated optical density) was selected as the measurement. The intensity of CN + CC was determined as 100% for Cerulean, and the intensity of VN + CC was determined as 100% for Venus.

### Western blot

Cells were collected 48 h after transfection using buffer containing 20 mM Tris pH 8.0, 200 mM NaCl, 1% Triton-X100, 2 mM DTT, 5 mM 4-nitrophenyl phosphate di (Tris) salt, 2 mM Na_2_VO_4_, and 1 mM PMSF supplemented with protease inhibitor cocktail (sigma). The same amount of samples was separated by 10% SDS-PAGE and transferred to nitrocellulose membrane (Bio-Rad, 162–0112). Immunodetection was performed by specific antibodies. Signals were captured by using the ChemiDoc imaging system (Bio-Rad ChemiDoc MP).

### Luciferase reporter assay

HeLa cells were transiently transfected with Flag-p52 (1–415), Flag-Bcl3(1–446), Flag-RelB (1–559), and luciferase reporter vector with specific κB site sequences. The total amount of plasmid DNA was kept constant for all assays. Cells were collected 48 h after transfection. Luciferase activities were measured using the dual-luciferase reporter assay system (Promega) following the manufacturer’s instructions. Briefly, cells were lysed by 1X Passive Lysis Buffer (PLB) and mixed with Luciferase Assay Reagent II (LAR II) in a 96-well plate, the luciferase activity was then measured by Plate-Reading Luminometers (PerkinElmer Victor X3).

### Co-immunoprecipitation

The whole cell lysates with indicated plasmids transfections were pre-cleared by Protein A PLUS agarose bead and immunoprecipitated with the indicated antibody at 4°C overnight in the binding buffer (20 mM Tris pH 7.5, 150 mM NaCl, 0.5% NP-40, 1 mM DTT). The immunocomplexes were precipitated by Protein A PLUS agarose bead and subjected to Western blot. p52, RelB, Bcl3, p50, and RelA were detected using anti-p52, anti-RelB, anti-Bcl3, anti-p50, anti-RelA, anti-Flag, and anti-HA antibodies.

### Correlation analysis from The Cancer Genome Atlas and the Genotype-Tissue Expression databases

Breast invasive carcinoma (TCGA-BRCA), brain lower grade glioma (TCGA-LGG), lung adenocarcinoma (TCGA-LUAD), thyroid carcinoma (TCGA-THCA), and ovarian serous cystadenocarcinoma (TCGA-OV) transcriptome data (raw gene read counts) were downloaded from GDC (http://portal.gdc.cancer.gov/projects/). Also breast, brain, lung, and thyroid tissue expression data were downloaded from GTEx (https://gtexportal.org/home/). Data were assembled into a matrix using R package TCGA biolinks (Colaprico et al., 2016). Subsequently, TCGA/GTEx read count data containing 56,499/51,555 genes, respectively, with raw reads were pre-processed by filtering out genes with zero read counts across different samples within the cohort. After filtering, 23,183/35,813 genes remained per sample in TCGA/GTEx cohort. The read count data were normalized to produce transcripts per kilobase million (TPM—counts per length of transcript (kb) per million reads mapped), then normalized to log2 transformed transcripts per kilobase million (log2 (TPM)), followed by quantile normalization which can be used for downstream analysis.

The specific two genes’ correlation coefficients (R) were calculated by the Pearson’s correlation. The *p*-value was adjusted by using the Benjamini and Hochberg false discovery rate (FDR) method. All the analyses were performed in R 3.6.1 (https://www.r-project.org/).

### Protein expression and purification

Recombinant non-tagged human p52 (1–398) and p50 (1–435) was expressed and purified from *Escherichia coli* Rosetta (DE3) cells. Rosetta (DE3) cells transformed with pET-11a-p52 (1–398) or pET-11a-p50 (1–435) were cultured in 2 L of LB medium containing 50 mg/ml ampicillin and 34 mg/ml chloramphenicol at 37°C. Expression was induced with 0.2 mM isopropyl β-D-1-thiogalactopyranoside (IPTG) at OD_600_ 0.5–0.6 for 3 h. Cells were harvested by centrifugation, suspended in 40 mM Tris-HCl (pH 7.5), 100 mM NaCl, 10 mM β-mercaptoethanol (β-ME), 1 mM PMSF, and lysed by sonication. Cell debris was removed by centrifugation (20,000 g for 30 min). The clarified supernatant was loaded onto Q-Sepharose FF column (GE Healthcare). Flow-through fraction was applied to SP HP column (GE Healthcare). The column was washed with buffer containing 40 mM Tris-HCl (pH 7.5), 200 mM NaCl, 10 mM β-ME; and the protein was eluted by the same buffer containing 400 mM NaCl. Then the protein was concentrated and loaded onto the gel filtration column (HiLoad 16/600 Superdex 200 pg, GE Healthcare) pre-equilibrated with 10 mM Tris-HCl (pH 7.5), 100 mM NaCl, and 5 mM β-ME. Peak fractions were concentrated to ∼10 mg/ml, flash frozen in liquid nitrogen, and stored at −80°C. His-Bcl3(1–446) was expressed in *Escherichia coli* Rosetta (DE3) cells by induction with 0.2 mM IPTG at OD_600_ 0.4 for 8 h at 24°C. Cell pellets of 2 L-culture of Bcl3 were resuspended together in buffer containing 20 mM Tris-HCl (pH 8.0), 300 mM NaCl, 25 mM imidazole, 10% glycerol, 10 mM β-ME, 0.1 mM PMSF, and 50 μl Protease Inhibitor Cocktail (Sigma) and then purified by Ni Sepharose (HisTrap HP, GE), followed by an anion exchange column (Q Sepharose fast flow, GE). The Bcl3 protein further went through the HiTrap Desalting Column (GE) to exchange buffer before BLI assays.

### Biolayer interferometry assays

The kinetic assays were performed on the Octet K2 (ForteBio) instrument at 20°C with shaking at 1000 RPM. The Ni-NTA biosensors were used for protein–protein interactions and were hydrated in BLI buffer containing 20 mM Tris-HCl (pH 8.0), 200 mM NaCl, 5% glycerol, 1 mM DTT, and 0.02% (v/v) Tween-20. His-tagged-Bcl3 was loaded at 500 μg/ml for 90 s prior to baseline equilibration for 180 s in the BLI buffer. Association of p52 or p50 in BLI buffer at various concentrations was carried out for 240 s prior to dissociation for 360 s. All data were baseline subtracted and analyzed in ForteBio data analysis software using a global fitting to a 1:1 binding model. The experiments were performed in duplicate.

### Generation of stable cell lines overexpressing p52 or Bcl3

Human p52 (1–415) or Bcl3 (1–446) was cloned into the pLV vector. pLV-p52 or pLV-Bcl3 were transfected to HEK 293T cells together with packaging and envelope plasmids MDL, VSVG, and Rev. Two days after transfection, viral supernatants were harvested and filtered through the 0.45 μm filter. A2780 cells were infected with viral supernatants in the presence of 10 μM polybrene (Millipore, TR-1003-G) for 2 days. pLV-EGFP were used as infection control. The expressions of p52 or Bcl3 and EGFP were confirmed by Western blot.

### RNA isolation and RT-qPCR

The total RNA was isolated using the RNeasy Mini kit (QIAGEN, 74,106), and it was reverse transcribed into cDNA using the PrimeScript™ II 1st strand cDNA Synthesis kit (TaKaRa). RT-qPCR was performed using the SYBR Green Master Mix (Applied Biosystems). Expression values were normalized to GAPDH. The sequences of the primers used for RT-qPCR are listed in Table 1.

**TABLE 1:** The sequences of the primers used for RT-qPCR.

Primer name	Primer sequence 5'→3′
Human *BCL3* forward	AAC​CTG​CCT​ACA​CCC​CTA​TAC
Human *BCL3* reverse	CAC​CAC​AGC​AAT​ATG​GAG​AGG
Human *GAPDH* forward	GTC​TCC​TCT​GAC​TTC​AAC​AGC​G
Human *GAPDH* reverse	ACC​ACC​CTG​TTG​CTG​TAG​CCA​A
Human *MCP-1* forward	GAT​CTC​AGT​GCA​GAG​GCT​CG
Human *MCP-1* reverse	TGG​GGT​CAG​CAC​AGA​TCT​CC
Human *A20* forward	ACC​CCA​TTG​TTC​TCG​GCT​AT
Human *A20* reverse	AAT​CTT​CCC​CGG​TCT​CTG​TT
Human *ELC* forward	AGA​CGA​AGT​GGA​GAA​GTT​GAT​G
Human *ELC* reverse	ACG​AGG​TTT​AGC​TGG​ACA​TG
Human *BLC* forward	CCC​TAG​ACG​CTT​CAT​TGA​TCG
Human *BLC* reverse	ATT​CAG​CTT​GAG​GGT​CCA​C
Human *SDF-1* forward	AGA​GCC​AAC​GTC​AAG​CAT​C
Human *SDF-1* reverse	TGA​ATC​CAC​TTT​AGC​TTC​GGG
Human *CCND1* forward	ACT​ACC​GCC​TCA​CAC​GCT​TC
Human *CCND1* reverse	TTG​ACT​CCA​GCA​GGG​CTT​CG
Human *IL10* forward	GCC​TTT​AAT​AAG​CTC​CAA​GAG​AAA​GGC
Human *IL10* reverse	CGT​ATC​TTC​ATT​GTC​ATG​TAG​GCT​TC
Human *IL6* forward	ACA​GAT​TTG​AGA​GTA​GTG​AGG​A
Human *IL6* reverse	TCT​AGA​TTC​TTT​GCC​TTT​TTC​TG
Human *SELP* forward	GCA​TGC​AGA​GCT​GTG​AAA​TG
Human *SELP* reverse	GAT​CCA​TAA​CTG​AAG​TTT​CCC​C
Human *SKP2* forward	GAA​ACT​TTA​CTT​GAA​CTT​GGA
Human *SKP2* reverse	TCC​TTT​AAC​AGT​TGA​AGG​GT

## Results

### Expressions of *NFKB2*, *BCL3,* and *RELB* in cancer cells

**FIGURE 1:**
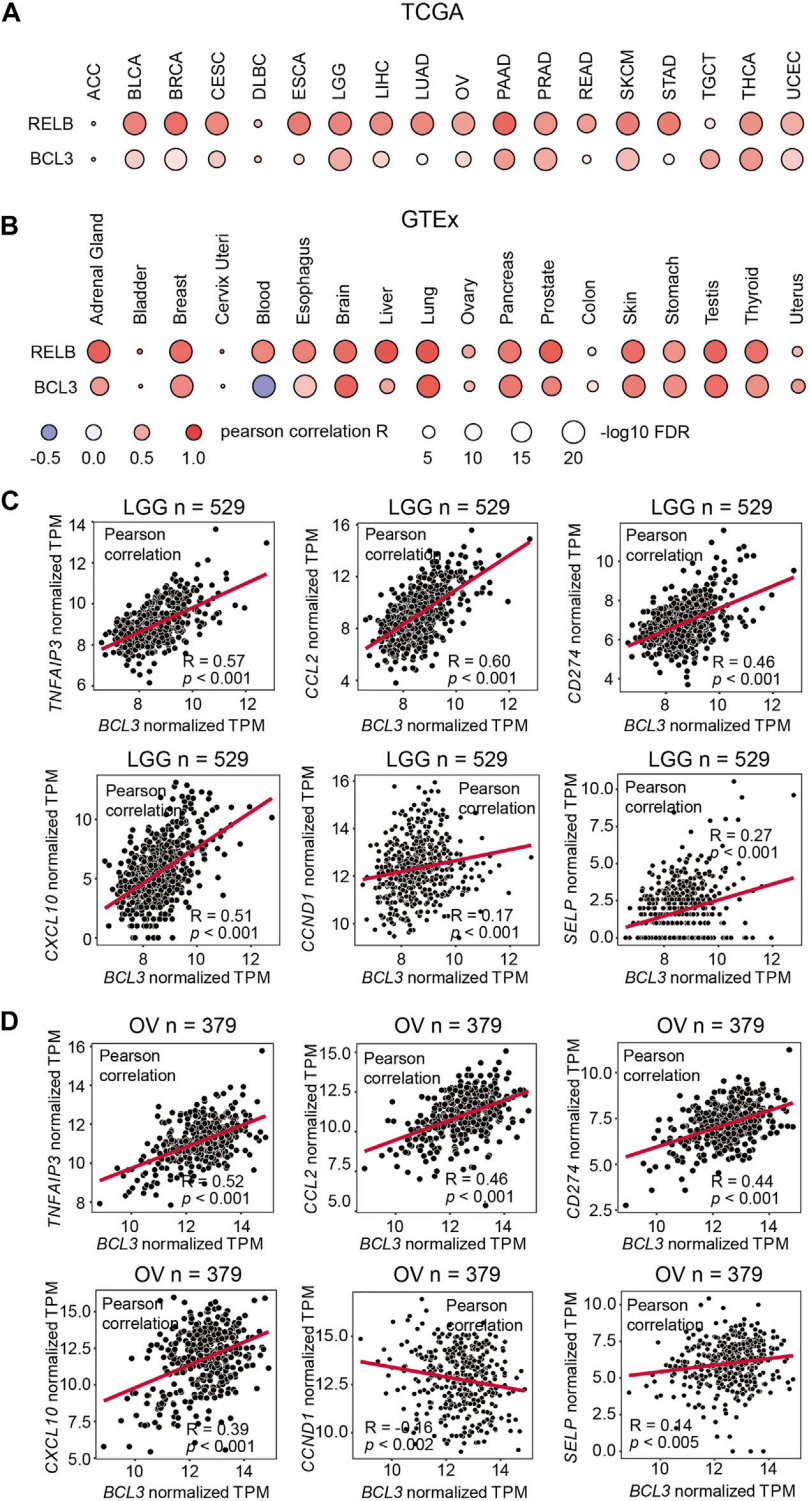
NFKB2 and RELB expressions are positively correlated. Correlation between *NFKB2*, *RELB,* and *BCL3* mRNA expressions in **(A)** various TCGA cancer types and **(B)** various GTEx tissue samples. Pearson correlation coefficient value (R) is shown by the blue-white-red color. R > 0 indicates the positive correlation; R < 0 indicates the negative correlation. The size of circles represents the values of log10 FDR. The abbreviation of each TCGA cancer type is listed on the following website: https://gdc.cancer.gov/resources-tcga-users/tcga-code-tables/tcga-study-abbreviations. Scatter plots showing the correlation between mRNA expressions of *BCL3* and p52:p52:Bcl3 target genes (*TNFAIP3*, *CCL2*, *CD274*, *CXCL10*, *CCND1*, and *SELP*) in **(C)** brain lower grade glioma (LGG) and **(D)** ovarian serous cystadenocarcinoma (OV) from TCGA database. “n” denotes the sample number.

NF-κB dimerization is affected by the dimer affinity as well as the subunit availability in cells. The defining event of the non-canonical NF-κB signaling is the processing of p100 into p52. However, the p52:RelB heterodimer is the predominant, if not the sole, NF-κB dimer is generated in this signaling. Indeed, p100 and RelB protein levels are positively correlated in mouse embryonic fibroblasts (MEFs) (Fusco et al., 2008; Fusco et al., 2016). The mechanism of how the p52:p52 homodimer is formed thus has remained unclear. Like RelB, Bcl3 also associates with p52, but unlike RelB which heterodimerizes with p52, Bcl3 forms a trimeric complex with the p52:p52 homodimer. As the processed product of p100, the cellular p52 level is affected by the expression level of p100. In order to investigate the *NFKB2*, *RELB,* and *BCL3* mRNA expression levels in a broad range, a correlation analysis was performed using both The Cancer Genome Atlas (TCGA) database and the Genotype-Tissue Expression (GTEx) database, which shows the mRNA expression from cancer tissues and normal tissues, respectively. A strong positive correlation was shown for *NFKB2* and *RELB* in most of the samples; however, only weak correlation was found between *NFKB2* and *BCL3* expression levels in the TCGA samples and somewhat stronger in the GTEx samples (Figures 1A,B). These information suggested that Bcl3 does not regulate NF-κB2/p100 at the mRNA expression level; other mechanisms might be involved in regulating p52:p52 dimer formation.

We also explored the correlation of Bcl3 and p52 target gene expressions in cancer samples. Bcl3 has been shown to contribute to tumorigenesis in glioma (Wu et al., 2018) and ovarian cancer (Zou et al., 2018). Thus, we examined the correlation of target genes in these two cancer types. Tumor necrosis factor alpha-induced protein 3 (*TNFAIP3/A20*), *CCL2*, *CD274*, *CXCL10,* Cyclin D1 (*CCND1*), and P-selectin (*SELP*) were reported to be regulated by the p52:p52:Bcl3 complex (Pan and McEver, 1995; Guttridge et al., 1999; Cogswell et al., 2000; Wang et al., 2012; Zou et al., 2018). Positive correlations were found between the mRNA levels of *BCL3* with some of these target genes, such as *TNFAIP3/A20*, *CCL2*, *CD274,* and *CXCL10,* in brain lower grade glioma (LGG) and ovarian serous cystadenocarcinoma (OV) samples from TCGA database (Figures 1C,D; Supplementary Figures S1B,C). These genes are also known to be regulated by other NF-κB family members, such as RelA; and they usually have high expression levels due to their multiple κB sites in the promoter/enhancer region: *TNFAIP3/A20* contains (-39) GGGTTATTCCC, (-54) GGAAATCCC, (-66) GGAAAGTCCC, and (-2188) GGGTACTTCC (Krikos et al., 1992; Wang et al., 2012); *CCL2* contains (-84) GGAAGATCCC, (-2610) GGGAATTTCC, and (-2638) GGGAACTTCC (Martin et al., 1997; Ueda et al., 1997); *CD274* contains (-542) GGGCTACCCC and (-9883) GGGAAGATCC; and *CXCL10* contains (-113) GGGACTTCCC and (-169) GGGAAATTCC (Leung et al., 2004). However, the analyses showed poor correlation in the case of *CCND1* and *SELP*, which are bona fide p52:p52:Bcl3 target genes. These two genes have a single κB site to be specifically regulated by p52, but not other NF-κB subunits: *CCND1* (-39) GGGGAGTTTT (Guttridge et al., 1999) and *SELP* (-218) GGGGTGACCC (Pan and McEver, 1995). Lack of or weak expression correlation between *BCL3* and *NFKB2* with these two genes is not indicative of the absence of the p52:p52 complex, but their amounts might be lower in most cell types tested earlier.

### Bcl3 stabilizes the p52:p52 homodimer by competing with RelB

We further explored the role of Bcl3 in p52:p52 homodimer formation using the BiFC assay. BiFC is an assay which tests protein–protein interactions in living cells, and it has been widely used in various model organisms. The principle of BiFC is that a fluorescent protein is divided into two non-fluorescent fragments and are fused with proteins of interest that might interact. The interaction of two target proteins brings the non-fluorescent fragments together which reconstitute into an intact fluorescent protein (Hu et al., 2006). In addition, multicolor BiFC could be used to detect three proteins’ competition (Hu and Kerppola, 2003; Shyu et al., 2006). Here, the multicolor BiFC assays were used to visualize different NF-κB dimers formation in living cells. The N-terminal fragment of fluorescent protein Venus (VN) and the N- or C-terminal fragments of Cerulean (CN or CC) were fused to the C-terminus of p52 or RelB RHR, named as p52-VN, p52-CN, RelB-VN, RelB-CN, and RelB-CC (Supplementary Figure S2A). Co-expression of p52-VN with RelB-CC, or p52-CN with RelB-CC in HEK 293T cells generated strong fluorescent signals in Venus and Cerulean channels, indicating the formation of the p52:RelB heterodimer (Supplementary Figures S2B,C). However, co-expression of neither RelB-VN with RelB-CC nor RelB-CN with RelB-CC gave any fluorescent signals, indicating RelB failed to form homodimer in cells which is in line with the literature (Vu et al., 2013). This observation also confirms that dimerization does not occur artificially through the association of the two complementary fragments of the reporter protein and that it truly measures preferential association of two NF-κB subunits.

**FIGURE 2:**
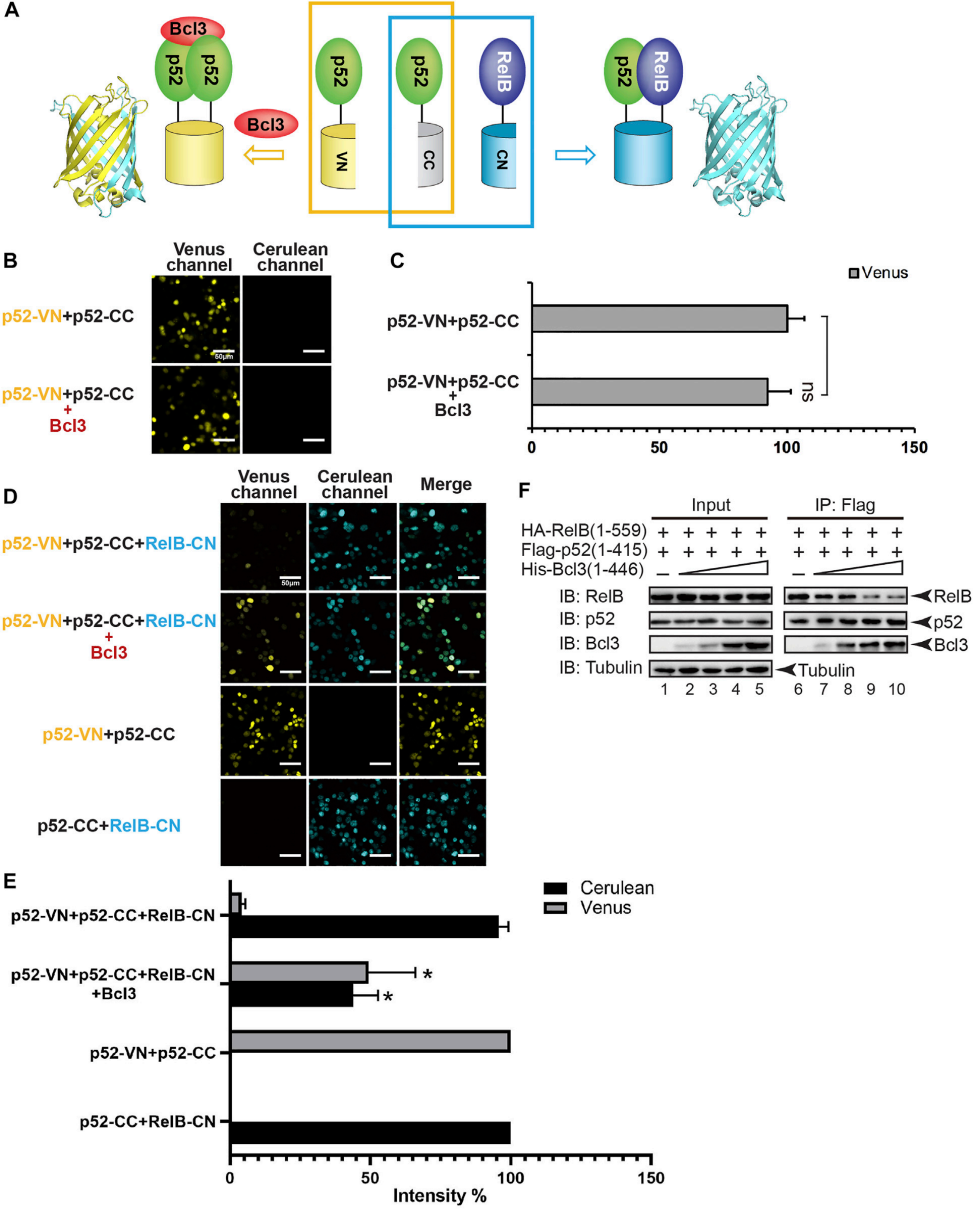
Bcl3 enhances the generation of the p52:p52 homodimer: p52:p52 vs. p52:RelB. **(A)** Diagram illustrating the principle of the multicolor BiFC assay. The p52:p52 homodimer formation brings VN and CN together to form a fluorescent protein which could be detected at the Venus channel; the p52:RelB heterodimer formation brings CC and CN together to form an intact Cerulean protein which could be detected using the Cerulean channel. **(B)** Fluorescent images with the co-expression of indicated plasmids. The addition of Bcl3 did not further enhance the fluorescence intensity of p52-VN and p52-CC. **(C)** Quantification of the fluorescence intensity in **(B)**; the data were analyzed from images of three independent experiments. n.s., not significant versus no Bcl3 co-expression (*t* test). Error bars represent standard deviation (SD). **(D)** Fluorescent images with the co-expression of indicated plasmids. The addition of Bcl3 enhances p52:p52 homodimerization. **(E)** Quantification of the fluorescence intensity in **(D)**, the fluorescence intensity of p52-VN + p52-CC was determined as 100% for Venus, and the fluorescence intensity of p52-CC + RelB-CN was determined as 100% for Cerulean. The data were analyzed from images of three independent experiments. *p < 0.05 versus no Bcl3 co-expression (*t* test). Error bars represent SD. **(F)** Co-IP showing less amount of p52 heterodimerized with RelB when there is an increasing amount of Bcl3 co-expressed.

To monitor the effect of Bcl3 on dimer formation, plasmids encoding p52-VN, p52-CC, and RelB-CN, with or without Bcl3 were co-transfected into HEK 293T cells (Figure 2A). The formation of p52:p52 homodimer and p52:RelB heterodimer could be detected by Venus and Cerulean fluorescent signals, respectively. The addition of Bcl3 to p52-VN and p52-CC did not further enhance the Venus fluorescence signal (Figures 2B,C; Supplementary Figure S2D); therefore, the fluorescent intensity of p52-VN + p52-CC was determined as 100% for Venus for the rest of the study. However, the expression level of p52-VN and p52-CC is likely higher than the dissociation constant (K_d_) of p52:p52 homodimer which might eliminate any effect of Bcl3 to stabilize the homodimer. Fluorescent images showed that in the absence of Bcl3, the p52:RelB heterodimer was the predominant dimer formed in cells; however, more p52:p52 homodimer was detected in the presence of Bcl3 (Figure 2D). The expression of Bcl3 did not affect either p52 or RelB expression levels (Supplementary Figure S2E). These results suggested that Bcl3 could reserve a pool of p52:p52 homodimer in cells without affecting its protein expression level.

Since NF-κB proteins are obligate dimers and p52 primarily heterodimerizes with RelB, we speculated that the increase of p52:p52 homodimerization by Bcl3 could lead to the decrease of p52:RelB heterodimer formation. Constant amount of Flag-p52 and HA-RelB, together with increasing amount of His-Bcl3 were co-transfected into HEK 293T cells. Flag-p52 immunoprecipitation (IP) showed that less RelB heterodimerized with p52 when there is increasing amount of Bcl3. Meanwhile, increasing amount of Bcl3 was detected to interact with p52 when more Bcl3 was transfected into cells (Figure 2F; Supplementary Figure S2F). Since Bcl3 is known to interact with p52:p52 homodimer but not p52:RelB heterodimer (Bours et al., 1993; Inoue et al., 1993), these results indicated that more p52:p52 homodimer was formed in a dose-dependent manner when there is more Bcl3.

We further quantitated the dimer distribution by measuring the intensity of Venus and Cerulean fluorescence. The fluorescent intensity of p52-VN + p52-CC was set as 100% for Venus; and the fluorescent intensity of p52-CC + RelB-CN was set as 100% for Cerulean. In the absence of Bcl3, the p52:RelB heterodimer was estimated to be ∼20-fold more stable than the p52:p52 homodimer (Figure 2E). However, the Bcl3 alters the balance by preferentially stabilizing the homodimer resulting in near equal distribution of both dimers.

### Bcl3 effectively competes with all other NF-κB subunits for p52 binding

**FIGURE 3:**
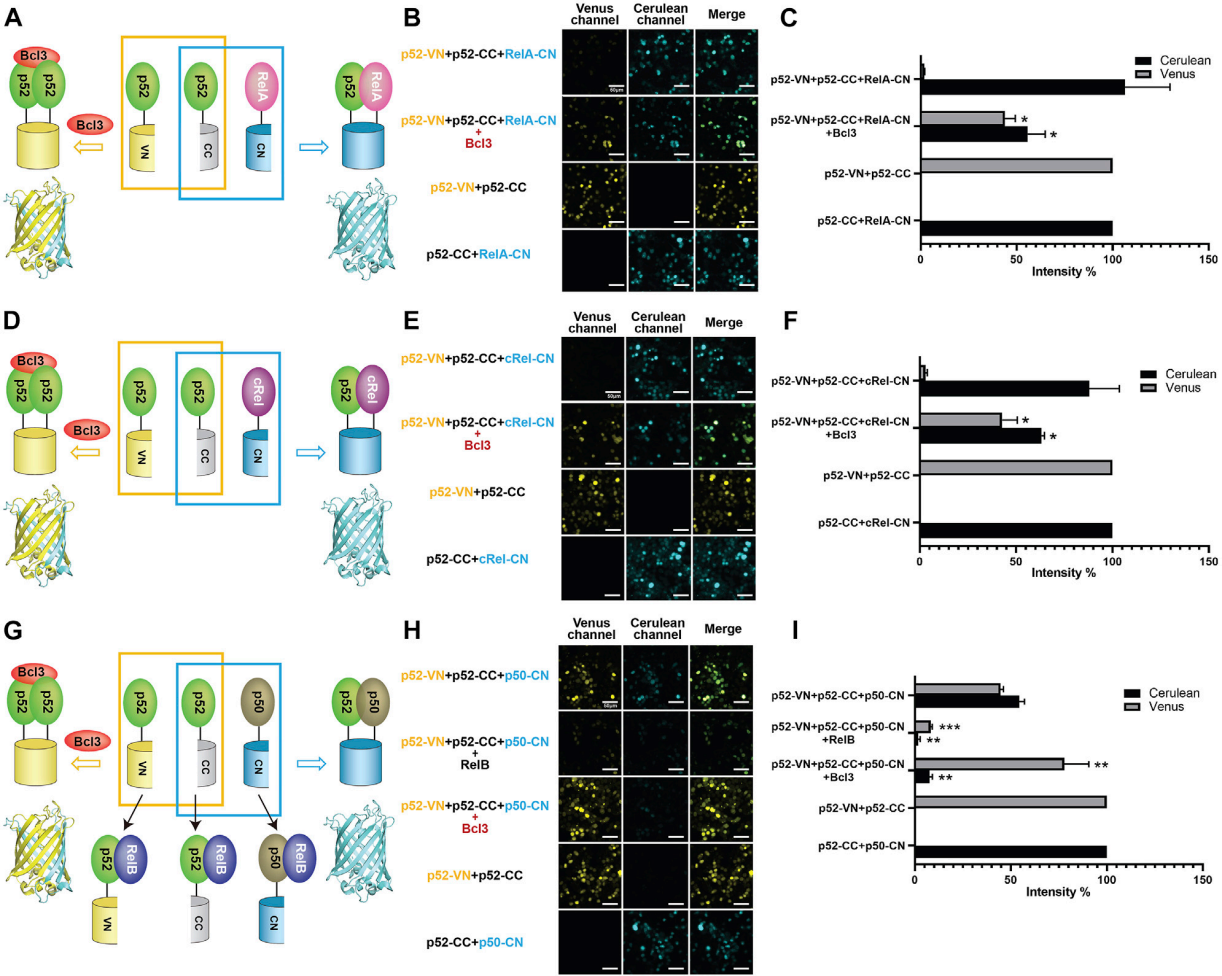
Bcl3 enhances the generation of the p52:p52 homodimer: p52:p52 vs. p52:RelA/p52:cRel/p52:p50. Diagrams illustrating the principle of multicolor BiFC assays: the p52:p52 homodimer formation brings VN and CN together to form a fluorescent protein which could be detected at the Venus channel; the **(A)** p52:RelA, **(D)** p52:cRel, and **(G)** p52:p50 heterodimer formation brings CC and CN together to form an intact Cerulean protein which could be detected using the Cerulean channel. The addition of RelB will heterodimerize with p52 or p50; therefore, both p52:p52 (Venus signal) and p52:p50 (Cerulean signal) will be decreased in **(G)**. Fluorescent images of **(B)** p52:p52 vs. p52:RelA, **(E)** p52:p52 vs. p52:cRel, and **(H)** p52:p52 vs. p52:p50. The addition of Bcl3 enhanced p52:p52 homodimerization in all cases, as shown by the enhanced Venus signals; the addition of RelB decreased both p52:p52 (Venus) and p52:p50 (Cerulean) heterodimers in **(H)**. **(C,F,I)** Quantification of the fluorescence intensity in **(B)**, **(E)**, and **(H)**. The fluorescence intensity of p52-VN + p52-CC was determined as 100% for Venus, and the fluorescence intensity of p52-CC + RelB-CN, p52-CC + cRel-CN, or p52-CC + p50-CN was determined as 100% for Cerulean. The data were analyzed from images of three independent experiments. *p < 0.05; **p < 0.01; ***p < 0.001 versus no Bcl3 or RelB co-expression (*t* test). Error bars represent SD.

The p52 protein could possibly form five NF-κB dimers: four heterodimers with four other NF-κB subunits and the p52:p52 homodimer. The p52:RelB and p52:RelA heterodimers are the most abundant p52 dimers *in vivo* and their physiological activities are well documented (Hoffmann et al., 2003; Basak et al., 2008). p52 may also form dimers with p50 and cRel; however, very little is known about their existence or cellular function. Using the multicolor BiFC system, we further examined the formation of other p52-NF-κB dimers and the effect of Bcl3 on these dimers. To study the generation of p52:RelA vs. p52:p52 dimers, plasmids encoding p52-VN, p52-CC, and RelA-CN were co-expressed in HEK 293T cells (Figures 3A; Supplementary Figure S3A). The results showed that p52:RelA heterodimers were also preferred over the p52:p52 homodimers in cells, and the presence of Bcl3 increased the p52:p52 homodimer levels (Figures 3B,C; Supplementary Figure S3B). Similar results were observed for cRel; p52 primarily heterodimerizes with cRel in the absence of Bcl3, and more p52:p52 homodimer was generated when Bcl3 was co-expressed (Figures 3D–F; Supplementary Figure S3C). Taken together, these observations suggest that p52 homodimer is significantly weaker than its heterodimers with RelA, RelB, and cRel.

Next, we investigated if p52 heterodimerizes with p50 in cells. p52-VN, p52-CC, and p50-CN were transfected into HEK 293T cells, both p52:p52 homodimer (Venus) and p52:p50 heterodimer (Cerulean) were seen. Similar fluorescent intensity suggests the dimer affinity of the p52:p50 heterodimer is comparable to the p52:p52 homodimer (Figures 3G–I; Supplementary Figure S3D). We then monitored the changes of these two dimers by co-expressing either RelB or Bcl3. When co-expressing RelB, amounts of both the p52:p52 and p52:p50 dimers decreased indicating the formation of p52:RelB and p50:RelB heterodimers is flavored in cells. When co-expressing Bcl3, again, the p52:p52 homodimer increased while the p52:p50 heterodimer decreased. This also suggests that there is no or only weak interaction between Bcl3 and p50:p52 heterodimer. In summary, our results showed that Bcl3 could successfully reserve a pool of the p52:p52 homodimer in competing with all other p52 containing dimers (p52:RelB, p52:RelA, p52:cRel, and p52:p50) in cells.

### Bcl3 does not stabilize the p50:p50 homodimer

**FIGURE 4:**
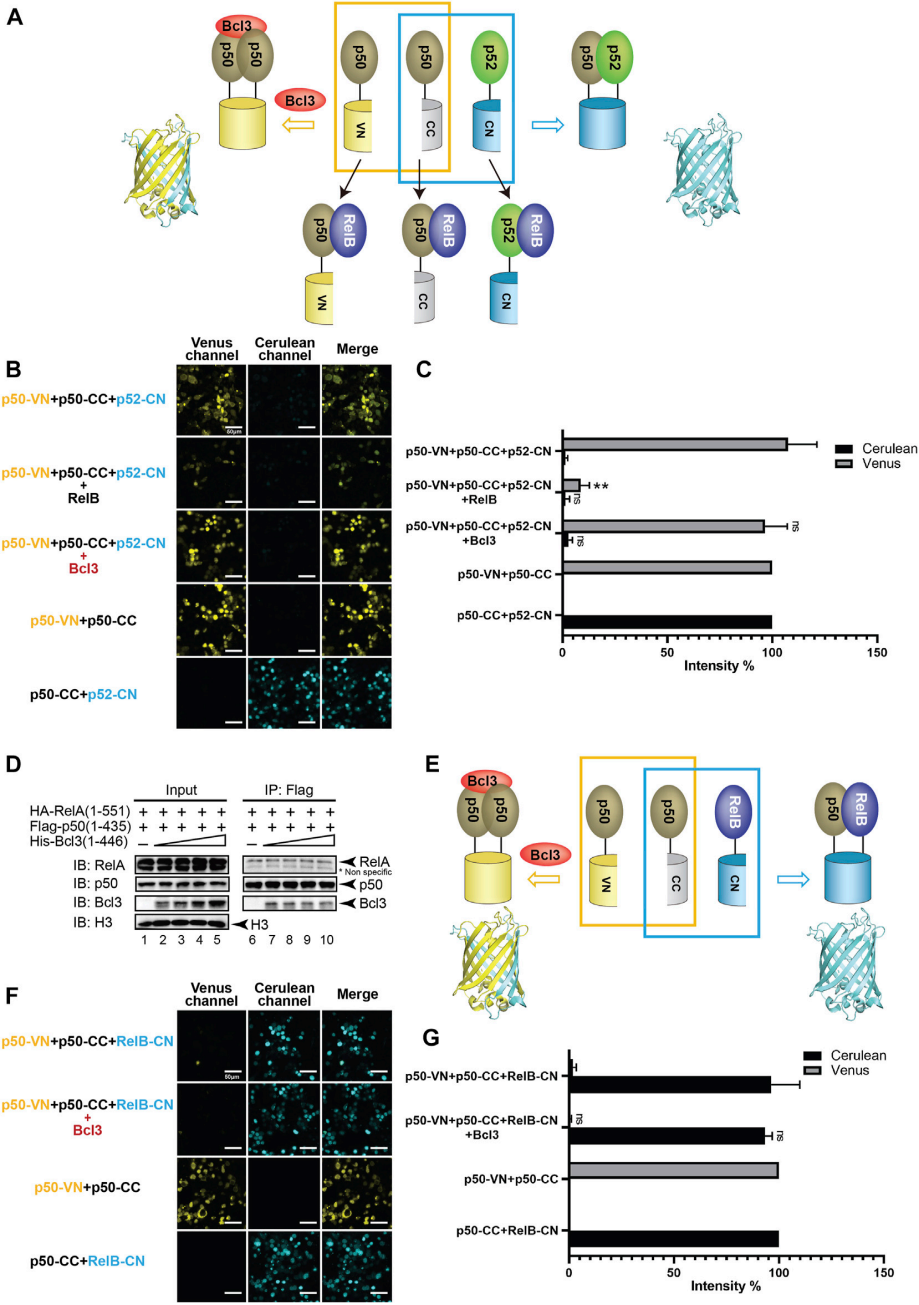
Bcl3 does not enhance the generation of the p50:p50 homodimer. **(A)** Diagram illustrating the principle of multicolor BiFC assay. The p50:p50 homodimer formation brings VN and CN together to form a fluorescent protein which could be detected at the Venus channel; the p52:p50 heterodimer formation brings CC and CN together to form an intact Cerulean protein which could be detected using the Cerulean channel. The addition of RelB will heterodimerize with p50 or p52; therefore, both p50:p50 (Venus) and p52:p50 (Cerulean) will be decreased. **(B)** Fluorescent images with the co-expression of indicated plasmids. The p50:p50 (Venus) is a stronger dimer than p52:p50 (Cerulean); and the addition of Bcl3 does not affect their dimer formation. **(C)** Quantification of the fluorescence intensity in **(B)**; the intensity of p50-VN + p50-CC was determined as 100% for Venus, and the fluorescence intensity of p50-CC + p52-CN was determined as 100% for Cerulean. The data were analyzed from images of three independent experiments. **p < 0.01; n. s., not significant versus no Bcl3 co-expression (*t* test). Error bars represent SD. **(D)** Co-IP showing the expression of Bcl3 does not affect the interaction of p50 and RelA. **(E)** Diagram illustrating the principle of multicolor BiFC assay. The p50:p50 homodimer formation brings VN and CN together to form a fluorescent protein which could be detected at the Venus channel; the p50:RelB heterodimer formation brings CC and CN together to form an intact Cerulean protein which could be detected using the Cerulean channel. **(F)** Fluorescent images with the co-expression of indicated plasmids. The addition of Bcl3 does not affect either p50:p50 (Venus) or p50:RelB (Cerulean) dimers. **(G)** Quantification of the fluorescence intensity in **(F)**; the intensity of p50-VN + p50-CC was determined as 100% for Venus, and the fluorescence intensity of p50-CC + RelB-CN was determined as 100% for Cerulean. The data were analyzed from images of three independent experiments. n. s., not significant versus no Bcl3 co-expression (*t* test). Error bars represent SD.

Selectivity against the p50 subunit in the p50:p52 heterodimer by Bcl3 contrasts previous reports (Carmody et al., 2007; Kabuta et al., 2010). Bcl3 was also known to interact with the p50:p50 homodimer in the NF-κB family (Fujita et al., 1993; Nolan et al., 1993), we would also like to explore if Bcl3 could enhance the p50:p50 homodimer population. Plasmids encoding p50-VN, p50-CC, and p52-CN were transfected into HEK 293T cells, the p50:p50 homodimer and p52:p50 heterodimer could be seen by Venus and Cerulean, respectively (Figure 4A; Supplementary Figure S4A). Strong Venus fluorescent intensity suggests that the p50:p50 homodimer is a stronger dimer than the p52:p50 heterodimer; the addition of Bcl3 did not further enhance the p50:p50 homodimerization (Figures 4B,C; Supplementary Figures S4B–E). The p50:RelA heterodimer is the most abundant NF-κB dimer in cells, the p50:p50 homodimer is a lower affinity dimer compared to the p50:RelA heterodimer (Tsui et al., 2015; Ramsey et al., 2019). The Flag-p50 IP assay was performed to monitor whether Bcl3 affects p50:p50 and p50:RelA dimer formation. Constant amount of Flag-p50 and HA-RelA, together with increasing amount of His-Bcl3 were co-expressed in HEK 293T cells. The Flag-p50 IP results showed that the amount of RelA interacting with p50 was not affected by the increasing amount of Bcl3 (Figure 4D). Since the p50:p50 homodimer is a stronger dimer than both the p52:p50 and p52:p52 dimers, Bcl3 might not further influence the p50:p50 homodimer formation. Similarly, the dimer affinity of p50:RelA might be stronger than the binding affinity between Bcl3 and p50; therefore, Bcl3 could not affect the formation of the p50:RelA heterodimer.

The p50 protein is also known to form a transcriptional active heterodimer with RelB (Jiang et al., 2002; Bhardwaj et al., 2015). In NF-κB2/p100-deficient cells, the activation of p50:RelB increased (Chatterjee et al., 2019). The absence of p52:RelB in NF-κB2/p100-deficient cells was partially compensated by the existence of p50:RelB (Lo et al., 2006; Basak et al., 2008). The HA-RelB IP results showed that RelB interacted with more p52 than p50 when co-expressed in HEK293T cells (Supplementary Figure S4F). We further applied the multicolor BiFC system to test the role of Bcl3 in p50:p50 and p50:RelB dimer generation (Figure 4E; Supplementary Figure S4A). Similar to p52, p50 also preferentially formed a heterodimer with RelB in cells (Figure 4F). But surprisingly the co-expression of Bcl3 did not affect the dimer populations of either the p50:p50 homodimer or the p50:RelB heterodimer (Figures 4F,G; Supplementary Figure S4G).

**FIGURE 5:**
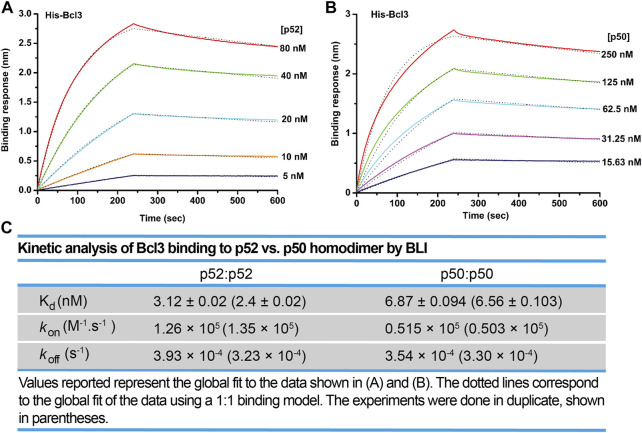
Bcl3 binds to p52 with higher affinity than p50. Biolayer interferometry (BLI) binding analysis of recombinant His-tagged-Bcl3 to purified **(A)** p52:p52 and **(B)** p50:p50 homodimers. Each experiment was performed in duplicate, and one representative set of curves is shown. **(C)** Table showing the kinetic analysis in **(A)** and **(B)**.

We next examined the binding affinity of Bcl3 with p52:p52 and p50:p50 homodimers using biolayer interferometry (BLI) assays. Recombinant His-tagged Bcl3 protein was immobilized on the Ni-NTA sensors and tested with purified p52 and p50 proteins (Supplementary Figures S5A,B). The binding affinity (revealed by the dissociation constant, K_d_) of Bcl3 with p52:p52 homodimer is more than 2-fold tighter than p50:p50 homodimer (Figures 5A–C). These results suggested that the role of Bcl3 in assisting p52:p52 homodimer generation is unique in the NF-κB family; and Bcl3 failed to enhance p50:p50 homodimer formation which is likely due to their lower binding affinity.

### Bcl3 increases the expression of p52:p52 homodimer target genes

**FIGURE 6:**
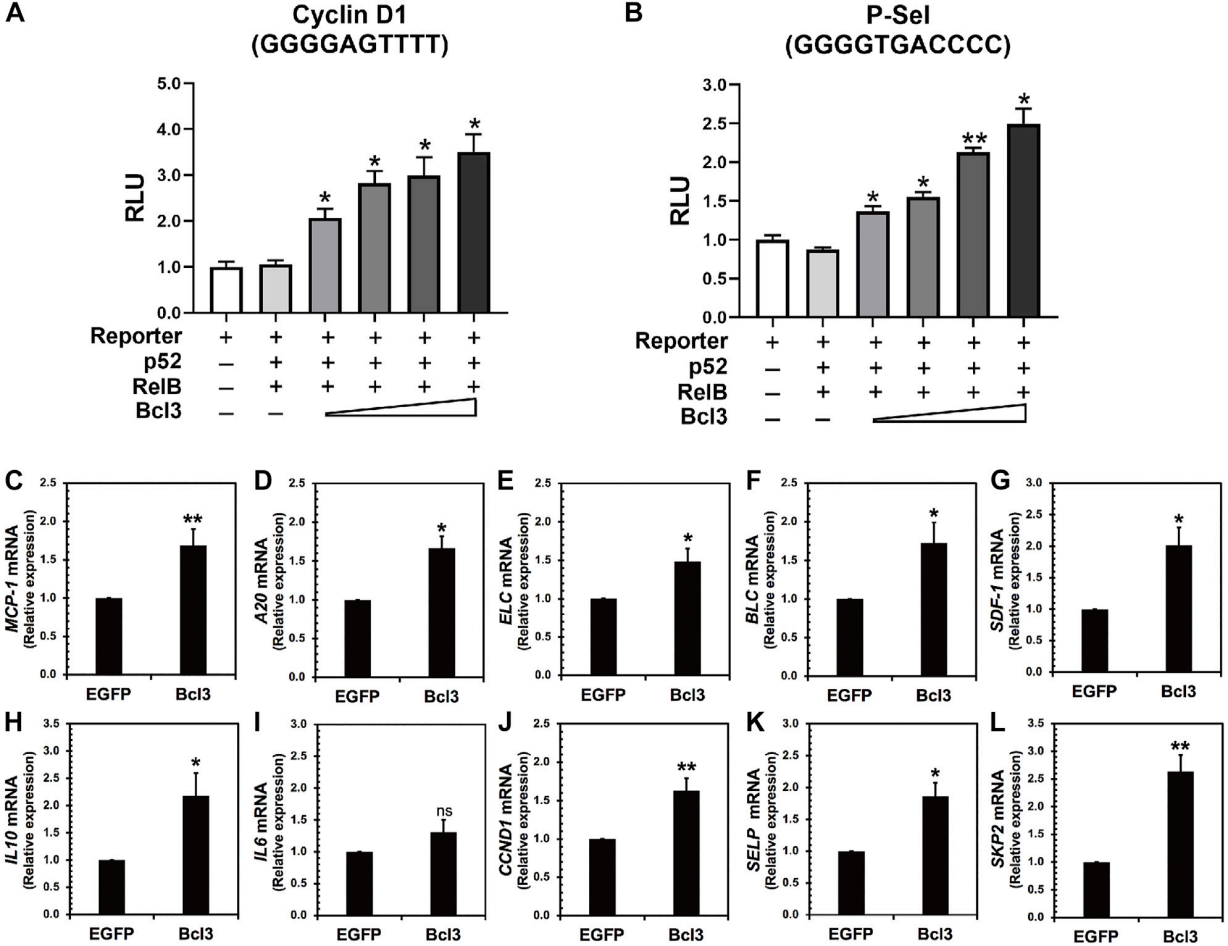
Bcl3 increases the expression of p52:p52 homodimer target genes. **(A)** P-selectin and **(B)** cyclin D1 luciferase reporter activities were increased with increasing Bcl3 co-expression. The reporter plasmid encoding the P-selectin or cyclin D1 κB site was co-transfected with a constant amount of p52, RelB, and an increasing amount of Bcl3 into HeLa cells. The data were analyzed from three independent experiments performed in triplicate. RLU, relative luciferase unit. *p < 0.05 and **p < 0.01 versus reporter only control (*t* test). Error bars represent SD. **(C–L)** Relative mRNA levels showing **(C)** *MCP-1*, **(D)** *A20*, **(E)** *ELC*, **(F)** *BLC*, **(G)** *SDF-1*, **(H)** *IL10*, **(I)** *IL6*, **(J)** *CCND1*, **(K)** *SELP,* and **(L)** *SKP2* were upregulated upon Bcl3 overexpression in A2780 cells by RT-qPCR analysis. *p < 0.05, **p < 0.01, n. s., not significant versus pLV-EGFP infection control (*t* test). Error bars represent SD.

The aforementioned results indicated that more p52:p52 homodimers could be found when Bcl3 protein levels increased in cells. Therefore, we hypothesized that the expression of specific target genes regulated by the p52:p52:Bcl3 complex would also be upregulated. NF-κB dimers activate gene transcription by binding to specific DNA response elements, termed κB sites or κB DNAs, present in promoter and/or enhancer regions of target genes (Mulero et al., 2019). By cloning the specific κB site of a target gene into the promoter of a luciferase reporter plasmid, the activation of the gene could be measured using luciferase activity by co-transfecting the reporter plasmid together with different NF-κB plasmids and co-factors. P-selectin and cyclin D1 are known target genes of the p52:p52:Bcl3 complex (Pan and McEver, 1995; Guttridge et al., 1999; Cogswell et al., 2000). HeLa cells were co-transfected with reporter plasmid encoding, either P-selectin or cyclin D1 κB site, together with constant amount of p52, RelB, and increasing amount of Bcl3. The p52:RelB heterodimer activates neither P-selectin nor cyclin D1 reporter; however, the addition of Bcl3 activates both reporters and the transcriptional activities increased with increasing amount of transfected Bcl3 (Figures 6A,B; Supplementary Figures S6A–C). As shown earlier, there were more p52:p52 homodimer generated with the increasing amount of Bcl3 (Figures 2D–F); therefore, these results suggested that Bcl3 enhanced the cellular p52:p52 homodimer population resulting in the upregulation of specific target genes regulated by the p52:p52:Bcl3 complex.

We further looked at several p52 target genes expression in ovarian cancer A2780 cells upon Bcl3 overexpression using lentiviral transduction (Figures 6C–L). The overexpression of Bcl3 did not affect either p52 or RelB protein levels in cells (Supplementary Figure S6D). The mRNA levels of monocyte chemoattractant protein-1 (*MCP-1*, also known as *CCL2*), *TNFAIP3/A20,* B lymphoblastoid cell chemokine (*BLC*), EBI1 ligand chemokine (*ELC*) and stromal cell-derived factor 1 (*SDF-1*), interleukin 10 (*IL10*), cyclin D1 (*CCND1*), P-selectin (*SELP*), and S-phase kinase-associated protein 2 (SKP2) were shown to be upregulated upon Bcl3 overexpression. Among these tested genes, *IL10* (with κB site GGGGCCTTCC), *CCND1* (GGGGAGTTTT), *SELP* (GGGGTGACCCC), and *SKP2* (GGGGAGTTCC) are specific targets for p52:p52:Bcl3. Interleukin 6 (*IL6*) has κB site GGGGATTTTCCC which might be preferentially regulated by the p50:p50 homodimer in cells; therefore, there was no significant change of *IL6* mRNA upon Bcl3 overexpression. Overall, our results suggested that Bcl3 effectively reserves a pool of p52:p52 homodimer in cells and consequently leads to the upregulation of p52:p52:Bcl3 target genes transcription.

## Discussion

In the dynamic cellular environment, the half-life of a protein is determined constantly by its synthesis and its degradation. Among other factors, folded stability of a protein and its ability to associate with various cellular factors determine their steady state levels. Since many proteins have common interactors, steady state levels of these complexes are determined by their relative strengths of association, competition with other factors, given that they are all available at time and space. In this study, we report a new and specific function for the atypical IκB protein Bcl3. NF-κB subunits associate with each other forming transcriptionally active dimers. It is known for a long time that p50:RelA heterodimer is more stable than the p50:p50 homodimer and that the RelA:RelA homodimer is the weakest among the three (Huang et al., 1997; Ramsey et al., 2019). Stability of these dimers is not only dictated by their dimerization affinity, but also by their ability to associate with IκB family proteins (Phelps et al., 2000; Ghosh et al., 2012). Hoffmann group showed that IκBβ stabilizes the RelA:RelA homodimer (Tsui et al., 2015). It was also reported that p50:p50 homodimer is stabilized by Bcl3 (Carmody et al., 2007). In this report, we tried to determine how p52:p52 homodimer is stabilized. Function of this homodimer is yet to be fully explored. Synthesis of p100, the precursor of p52, is mediated primarily by RelA dimers which is the product of the NF-κB canonical signaling; however, the maturation of p100 into p52 requires the non-canonical signaling (Ghosh and Wang, 2021). Since the NF-κB dimer generated by most known non-canonical signals is the p52:RelB heterodimer, it has remained an open question how the p52:p52 homodimer is formed.

**FIGURE 7:**
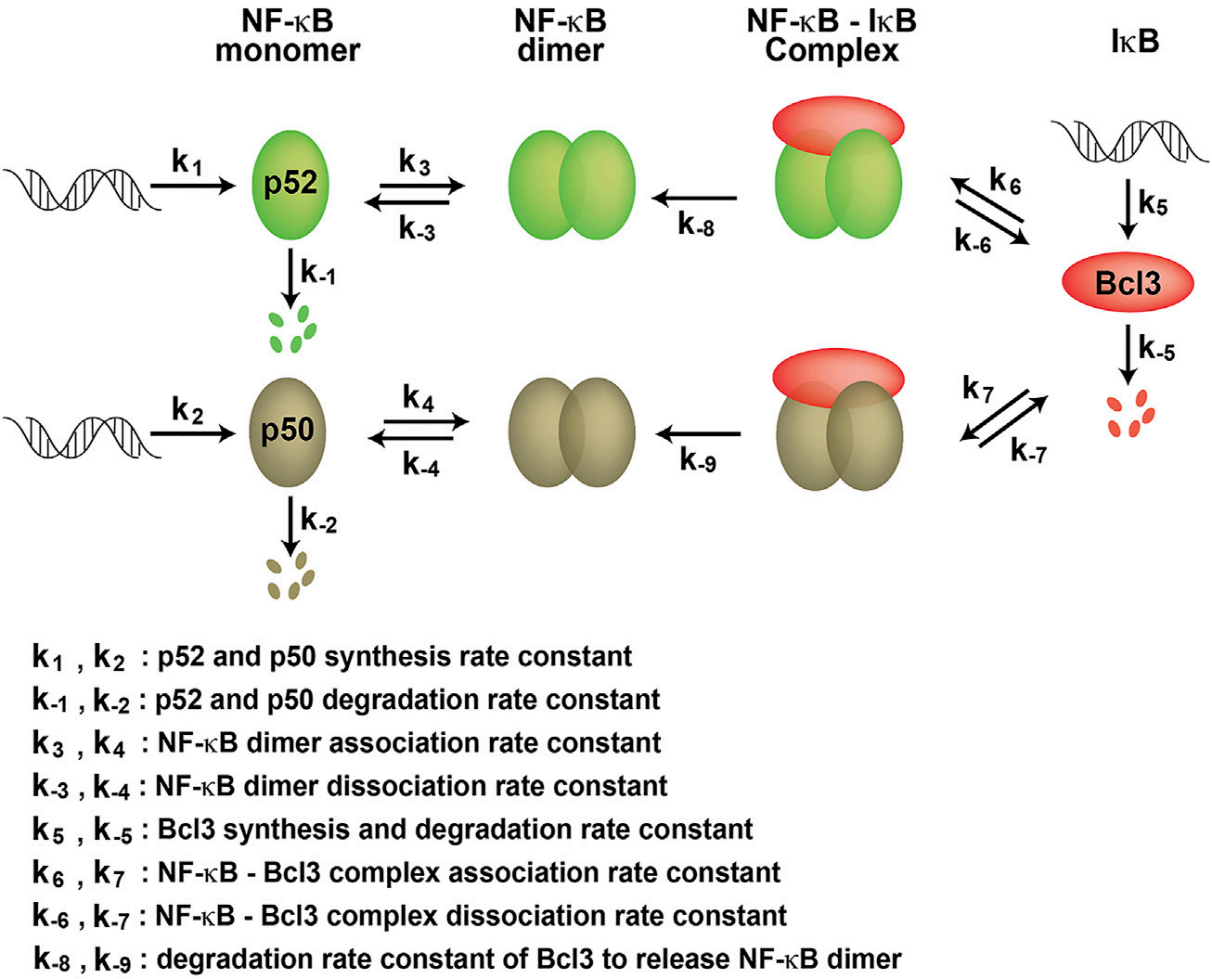
Kinetic model of NF-κB dimer generation. k_1_ and k_2_ are p52 and p50 synthesis rate constants; k_-1_ and k_-2_ are p52 and p50 degradation rate constants, respectively. k_3_ and k_4_ are dimer association rate constants, while k_-3_ and k_-4_ are p52:p52 and p50:p50 dimer dissociation rate constants, respectively. k_5_ and k_-5_ are the Bcl3 synthesis and degradation rate constants. k_6_ and k_7_ are the Bcl3-NF-κB complex association rate constants; k_-6_ and k_-7_ are the Bcl3-NF-κB complex dissociation rate constants. k_-8_ and k_-9_ are the degradation rate constants of Bcl3 to release NF-κB dimers.

We found that Bcl3 effectively competes with other NF-κB subunits for reserving a pool of p52:p52 homodimer in cells. This specific stabilization activity is unique to p52, not shared with other NF-κB subunits. Bcl3 binds to both p52 and p50 homodimers; however, its binding affinity for p52:p52 homodimer is more than 2-times higher than that of the p50:p50 homodimer. Interestingly, the affinity difference is mainly contributed by the rate of association (K_on_) but not by the rate of dissociation (K_off_). Bcl3 showed slightly higher association rate with p52:p52 (k_6_) than p50:p50 (k_7_) which suggested that p52 might be in a preferred conformation, making it easier for Bcl3 to collide and bind with p52. Cellular p50 is constitutively processed from its precursor protein p105, and the processed p50 mostly forms high affinity p50:RelA and p50:p50 dimers. However, the processing of p100 into p52 is a tightly regulated cellular process; only newly synthesized p100, but not pre-existing p100, can be processed into p52 and heterodimerized with the newly synthesized RelB (Mordmuller et al., 2003; Yilmaz et al., 2014). The newly synthesized p52 might be in a preferred conformation that Bcl3 could bind and stabilize its homodimerization. Once Bcl3 is bound to p52 or p50, the interactions are comparatively stable for both reflected by their similar dissociation rates (k_-6_ and k_-7_) (Figure 7). The NF-κB dimer affinity is determined by both its association and dissociation rates. As shown in Figures 3, 4, p52:p52 is a weaker dimer than p50:p50. The weaker p52:p52 dimer affinity could be possibly due to the association rate of p52:p52 (k_3_) being slower than the association rate of p50:p50 (k_4_). Alternatively, it could also be resulted from the fast dimer dissociation rate: p52:p52 and p50:p50 might have similar dimer association rates (k_3_ ≈ k_4_); however, p52:p52 might have a faster dissociation rate than that of p50:p50 (k_-3_ > k_-4_) resulting in less detectable p52:p52 homodimer in cells. Bcl3 might also play a role in the p52:p52 homodimerization by either increasing its association rate (k_3_) or decreasing its dissociation rate (k_-3_). Future studies are needed to fully understand the kinetics of dimer formation. Our observations suggest that if during the event of p100 processing into p52 and in the presence of Bcl3, some of p52 will be portioned into p52:p52 homodimer as a complex with Bcl3.

We further found that p52 heterodimers with RelA, RelB, and cRel are significantly more stable than the p52:p52 homodimer. Bcl3, however, stabilizes the homodimer even in the presence of these other NF-κB subunits. That is, if Bcl3 is present along with p52, RelA, RelB and cRel, p52 will be partitioned into four different dimers. The degree of portioning requires the knowledge of the rates of association and dissociation of these complexes, and the rates of synthesis and degradation of each subunit in cells.

We showed that in addition to the RelB homodimer, all other dimers can in principle form. The p52:RelB heterodimer formation is known to couple with the processing of p100 into p52 in the non-canonical NF-κB signaling (Fusco et al., 2008; Fusco et al., 2016). Therefore, even if the p52:RelA and p52:RelB heterodimers are of equal strengths, the p52:RelB heterodimer is more abundant. This also explains why p52:RelB is a more domain dimer over the p52:p52 homodimer. Nevertheless, Bcl3 can enhance the cellular population of p52:p52 homodimer. Future studies are needed to fully understand the detailed mechanism of these complexes’ formations *in vivo*.

Our work suggests that p52:p52:Bcl3 complex also serves as a repertoire for the p52:p52 homodimer which modulates specific downstream target genes’ transcription. This provides an explanation of *nfkb2* ^−/−^ or *bcl3* ^−/−^ mice exhibit similar defects, both are impaired in germinal centers formation and follicular dendritic cell networks (Franzoso et al., 1997; Schwarz et al., 1997; Franzoso et al., 1998; Paxian et al., 2002). It also provides additional evidence supporting the loss of both Bcl3 and NF-κB2, but not either one alone, severely compromises immunity (Zhang et al., 2007).

In sum, this study has revealed that Bcl3 is critical for the NF-κB p52:p52 homodimer. The p52 homodimerization is challenged in cells not only because of its relative weak dimer affinity, but also the competition with the higher affinity heterodimer partners, RelB, RelA, and cRel. Bcl3 might be able to bind p52 monomers sequentially so that it could compete with the bound heterodimer partners. Future studies are needed to uncover the detailed molecular mechanism of how Bcl3 preserves p52:p52 among all other dimers in the NF-κB family.

## Data Availability

The original contributions presented in the study are included in the article/supplementary material; further inquiries can be directed to the corresponding author.

## References

[B1] Basak S. Shih V. F. Hoffmann A. (2008). Generation and activation of multiple dimeric transcription factors within the NF-kappaB signaling system. Mol. Cell. Biol. 28, 3139–3150. 10.1128/MCB.01469-07 18299388 PMC2423155

[B2] Bhardwaj R. Yester J. W. Singh S. K. Biswas D. D. Surace M. J. Waters M. R. (2015). RelB/p50 complexes regulate cytokine-induced YKL-40 expression. J. Immunol. 194, 2862–2870. 10.4049/jimmunol.1400874 25681350 PMC4355396

[B3] Bours V. Franzoso G. Azarenko V. Park S. Kanno T. Brown K. (1993). The oncoprotein Bcl-3 directly transactivates through kappa B motifs via association with DNA-binding p50B homodimers. Cell 72, 729–739. 10.1016/0092-8674(93)90401-b 8453667

[B4] Carmody R. J. Ruan Q. Palmer S. Hilliard B. Chen Y. H. (2007). Negative regulation of toll-like receptor signaling by NF-kappaB p50 ubiquitination blockade. Science 317, 675–678. 10.1126/science.1142953 17673665

[B5] Chatterjee B. Roy P. Sarkar U. A. Zhao M. Ratra Y. Singh A. (2019). Immune differentiation regulator p100 tunes NF-κB responses to TNF. Front. Immunol. 10, 997. 10.3389/fimmu.2019.00997 31134075 PMC6514058

[B6] Chen F. E. Huang D. B. Chen Y. Q. Ghosh G. (1998a). Crystal structure of p50/p65 heterodimer of transcription factor NF-kappaB bound to DNA. Nature 391, 410–413. 10.1038/34956 9450761

[B7] Chen Y. Q. Ghosh S. Ghosh G. (1998b). A novel DNA recognition mode by the NF-kappa B p65 homodimer. Nat. Struct. Biol. 5, 67–73. 10.1038/nsb0198-67 9437432

[B8] Cogswell P. C. Guttridge D. C. Funkhouser W. K. Baldwin A. S. JR. (2000). Selective activation of NF-kappa B subunits in human breast cancer: Potential roles for NF-kappa B2/p52 and for bcl-3. Oncogene 19, 1123–1131. 10.1038/sj.onc.1203412 10713699

[B9] Colaprico A. Silva T. C. Olsen C. Garofano L. Cava C. Garolini D. (2016). TCGAbiolinks: An R/bioconductor package for integrative analysis of TCGA data. Nucleic Acids Res. 44, e71. 10.1093/nar/gkv1507 26704973 PMC4856967

[B10] Cramer P. Larson C. J. Verdine G. L. Muller C. W. (1997). Structure of the human NF-kappaB p52 homodimer-DNA complex at 2.1 A resolution. EMBO J. 16, 7078–7090. 10.1093/emboj/16.23.7078 9384586 PMC1170310

[B11] Fiorini E. Schmitz I. Marissen W. E. Osborn S. L. Touma M. Sasada T. (2002). Peptide-induced negative selection of thymocytes activates transcription of an NF-kappa B inhibitor. Mol. Cell 9, 637–648. 10.1016/s1097-2765(02)00469-0 11931770

[B12] Franzoso G. Carlson L. Poljak L. Shores E. W. Epstein S. Leonardi A. (1998). Mice deficient in nuclear factor (NF)-kappa B/p52 present with defects in humoral responses, germinal center reactions, and splenic microarchitecture. J. Exp. Med. 187, 147–159. 10.1084/jem.187.2.147 9432973 PMC2212099

[B13] Franzoso G. Carlson L. Scharton-Kersten T. Shores E. W. Epstein S. Grinberg A. (1997). Critical roles for the Bcl-3 oncoprotein in T cell-mediated immunity, splenic microarchitecture, and germinal center reactions. Immunity 6, 479–490. 10.1016/s1074-7613(00)80291-5 9133427

[B14] Fujita T. Nolan G. P. Liou H. C. Scott M. L. Baltimore D. (1993). The candidate proto-oncogene bcl-3 encodes a transcriptional coactivator that activates through NF-kappa B p50 homodimers. Genes Dev. 7, 1354–1363. 10.1101/gad.7.7b.1354 8330739

[B15] Fusco A. J. Huang D. B. Miller D. Wang V. Y. Vu D. Ghosh G. (2009). NF-kappaB p52:RelB heterodimer recognizes two classes of kappaB sites with two distinct modes. EMBO Rep. 10, 152–159. 10.1038/embor.2008.227 19098713 PMC2637311

[B16] Fusco A. J. Mazumder A. Wang V. Y. Tao Z. Ware C. Ghosh G. (2016). The NF-κB subunit RelB controls p100 processing by competing with the kinases NIK and IKK1 for binding to p100. Sci. Signal. 9, ra96. 10.1126/scisignal.aad9413 27678221

[B17] Fusco A. J. Savinova O. V. Talwar R. Kearns J. D. Hoffmann A. Ghosh G. (2008). Stabilization of RelB requires multidomain interactions with p100/p52. J. Biol. Chem. 283, 12324–12332. 10.1074/jbc.M707898200 18321863 PMC2431000

[B18] Ghosh G. VAN Duyne G. Ghosh S. Sigler P. B. (1995). Structure of NF-kappa B p50 homodimer bound to a kappa B site. Nature 373, 303–310. 10.1038/373303a0 7530332

[B19] Ghosh G. Wang V. Y. Huang D. B. Fusco A. (2012). NF-κB regulation: Lessons from structures. Immunol. Rev. 246, 36–58. 10.1111/j.1600-065X.2012.01097.x 22435546 PMC4543363

[B20] Ghosh G. Wang V. Y. (2021). Origin of the functional distinctiveness of NF-κB/p52. Front. Cell Dev. Biol. 9, 764164. 10.3389/fcell.2021.764164 34888310 PMC8650618

[B21] Guttridge D. C. Albanese C. Reuther J. Y. Pestell R. G. Baldwin A. S. JR. (1999). NF-kappaB controls cell growth and differentiation through transcriptional regulation of cyclin D1. Mol. Cell. Biol. 19, 5785–5799. 10.1128/mcb.19.8.5785 10409765 PMC84428

[B22] Hoffmann A. Leung T. H. Baltimore D. (2003). Genetic analysis of NF-kappaB/Rel transcription factors defines functional specificities. EMBO J. 22, 5530–5539. 10.1093/emboj/cdg534 14532125 PMC213788

[B23] Hu C. D. Grinberg A. V. Kerppola T. K. (2006). Visualization of protein interactions in living cells using bimolecular fluorescence complementation (BiFC) analysis. Curr. Protoc. Cell Biol. Chapter 21, Unit 21.3. Chapter 21Unit 21 3. 10.1002/0471143030.cb2103s29 18228482

[B24] Hu C. D. Kerppola T. K. (2003). Simultaneous visualization of multiple protein interactions in living cells using multicolor fluorescence complementation analysis. Nat. Biotechnol. 21, 539–545. 10.1038/nbt816 12692560 PMC1820765

[B25] Huang D. B. Huxford T. Chen Y. Q. Ghosh G. (1997). The role of DNA in the mechanism of NFkappaB dimer formation: Crystal structures of the dimerization domains of the p50 and p65 subunits. Structure 5, 1427–1436. 10.1016/s0969-2126(97)00293-1 9384558

[B26] Inoue J. Takahara T. Akizawa T. Hino O. (1993). Bcl-3, a member of the I kappa B proteins, has distinct specificity towards the Rel family of proteins. Oncogene 8, 2067–2073. 8336935

[B27] Jiang H. Y. Petrovas C. Sonenshein G. E. (2002). RelB-p50 NF-kappa B complexes are selectively induced by cytomegalovirus immediate-early protein 1: Differential regulation of bcl-x(L) promoter activity by NF-kappa B family members. J. Virol. 76, 5737–5747. 10.1128/jvi.76.11.5737-5747.2002 11992002 PMC137022

[B28] Kabuta T. Hakuno F. Cho Y. Yamanaka D. Chida K. Asano T. (2010). Insulin receptor substrate-3, interacting with Bcl-3, enhances p50 NF-kappaB activity. Biochem. Biophys. Res. Commun. 394, 697–702. 10.1016/j.bbrc.2010.03.054 20226764

[B29] Krikos A. Laherty C. D. Dixit V. M. (1992). Transcriptional activation of the tumor necrosis factor alpha-inducible zinc finger protein, A20, is mediated by kappa B elements. J. Biol. Chem. 267, 17971–17976. 10.1016/s0021-9258(19)37138-8 1381359

[B30] Leung T. H. Hoffmann A. Baltimore D. (2004). One nucleotide in a kappaB site can determine cofactor specificity for NF-kappaB dimers. Cell 118, 453–464. 10.1016/j.cell.2004.08.007 15315758

[B31] Lo J. C. Basak S. James E. S. Quiambo R. S. Kinsella M. C. Alegre M. L. (2006). Coordination between NF-kappaB family members p50 and p52 is essential for mediating LTbetaR signals in the development and organization of secondary lymphoid tissues. Blood 107, 1048–1055. 10.1182/blood-2005-06-2452 16195333 PMC1895903

[B32] Martin T. Cardarelli P. M. Parry G. C. Felts K. A. Cobb R. R. (1997). Cytokine induction of monocyte chemoattractant protein-1 gene expression in human endothelial cells depends on the cooperative action of NF-kappa B and AP-1. Eur. J. Immunol. 27, 1091–1097. 10.1002/eji.1830270508 9174597

[B33] Mathas S. Johrens K. Joos S. Lietz A. Hummel F. Janz M. (2005). Elevated NF-kappaB p50 complex formation and Bcl-3 expression in classical Hodgkin, anaplastic large-cell, and other peripheral T-cell lymphomas. Blood 106, 4287–4293. 10.1182/blood-2004-09-3620 16123212

[B34] Moorthy A. K. Huang D. B. Wang V. Y. Vu D. Ghosh G. (2007). X-ray structure of a NF-kappaB p50/RelB/DNA complex reveals assembly of multiple dimers on tandem kappaB sites. J. Mol. Biol. 373, 723–734. 10.1016/j.jmb.2007.08.039 17869269 PMC4167888

[B35] Mordmuller B. Krappmann D. Esen M. Wegener E. Scheidereit C. (2003). Lymphotoxin and lipopolysaccharide induce NF-kappaB-p52 generation by a co-translational mechanism. EMBO Rep. 4, 82–87. 10.1038/sj.embor.embor710 12524526 PMC1315810

[B36] Mulero M. C. Wang V. Y. Huxford T. Ghosh G. (2019). Genome reading by the NF-κB transcription factors. Nucleic Acids Res. 47, 9967–9989. 10.1093/nar/gkz739 31501881 PMC6821244

[B37] Nishikori M. Maesako Y. Ueda C. Kurata M. Uchiyama T. Ohno H. (2003). High-level expression of BCL3 differentiates t(2;5)(p23;q35)-positive anaplastic large cell lymphoma from Hodgkin disease. Blood 101, 2789–2796. 10.1182/blood-2002-08-2464 12456498

[B38] Nolan G. P. Fujita T. Bhatia K. Huppi C. Liou H. C. Scott M. L. (1993). The bcl-3 proto-oncogene encodes a nuclear I kappa B-like molecule that preferentially interacts with NF-kappa B p50 and p52 in a phosphorylation-dependent manner. Mol. Cell. Biol. 13, 3557–3566. 10.1128/mcb.13.6.3557 8497270 PMC359825

[B39] Ohno H. Doi S. Yabumoto K. Fukuhara S. Mckeithan T. W. (1993). Molecular characterization of the t(14;19)(q32;q13) translocation in chronic lymphocytic leukemia. Leukemia 7, 2057–2063. 8255106

[B40] Pan J. Mcever R. P. (1995). Regulation of the human P-selectin promoter by Bcl-3 and specific homodimeric members of the NF-kappa B/Rel family. J. Biol. Chem. 270, 23077–23083. 10.1074/jbc.270.39.23077 7559449

[B41] Paxian S. Merkle H. Riemann M. Wilda M. Adler G. Hameister H. (2002). Abnormal organogenesis of Peyer's patches in mice deficient for NF-kappaB1, NF-kappaB2, and Bcl-3. Gastroenterology 122, 1853–1868. 10.1053/gast.2002.33651 12055593

[B42] Phelps C. B. Sengchanthalangsy L. L. Huxford T. Ghosh G. (2000). Mechanism of I kappa B alpha binding to NF-kappa B dimers. J. Biol. Chem. 275, 29840–29846. 10.1074/jbc.M004899200 10882738

[B43] Ramsey K. M. Chen W. Marion J. D. Bergqvist S. Komives E. A. (2019). Exclusivity and compensation in NFκB dimer distributions and IκB inhibition. Biochemistry 58, 2555–2563. 10.1021/acs.biochem.9b00008 31033276 PMC6642826

[B44] Schwarz E. M. Krimpenfort P. Berns A. Verma I. M. (1997). Immunological defects in mice with a targeted disruption in Bcl-3. Genes Dev. 11, 187–197. 10.1101/gad.11.2.187 9009202

[B45] Shih V. F. Tsui R. Caldwell A. Hoffmann A. (2011). A single NFκB system for both canonical and non-canonical signaling. Cell Res. 21, 86–102. 10.1038/cr.2010.161 21102550 PMC3193412

[B46] Shyu Y. J. Liu H. Deng X. Hu C. D. (2006). Identification of new fluorescent protein fragments for bimolecular fluorescence complementation analysis under physiological conditions. Biotechniques 40, 61–66. 10.2144/000112036 16454041

[B47] Trinh D. V. Zhu N. Farhang G. Kim B. J. Huxford T. (2008). The nuclear I kappaB protein I kappaB zeta specifically binds NF-kappaB p50 homodimers and forms a ternary complex on kappaB DNA. J. Mol. Biol. 379, 122–135. 10.1016/j.jmb.2008.03.060 18436238

[B48] Tsui R. Kearns J. D. Lynch C. Vu D. Ngo K. A. Basak S. (2015). IκBβ enhances the generation of the low-affinity NFκB/RelA homodimer. Nat. Commun. 6, 7068. 10.1038/ncomms8068 25946967 PMC4425231

[B49] Ueda A. Ishigatsubo Y. Okubo T. Yoshimura T. (1997). Transcriptional regulation of the human monocyte chemoattractant protein-1 gene. Cooperation of two NF-kappaB sites and NF-kappaB/Rel subunit specificity. J. Biol. Chem. 272, 31092–31099. 10.1074/jbc.272.49.31092 9388261

[B50] Viatour P. Bentires-Alj M. Chariot A. Deregowski V. DE Leval L. Merville M. P. (2003). NF- kappa B2/p100 induces Bcl-2 expression. Leukemia 17, 1349–1356. 10.1038/sj.leu.2402982 12835724

[B51] Vu D. Huang D. B. Vemu A. Ghosh G. (2013). A structural basis for selective dimerization by NF-κB RelB. J. Mol. Biol. 425, 1934–1945. 10.1016/j.jmb.2013.02.020 23485337

[B52] Wang V. Y. Huang W. Asagiri M. Spann N. Hoffmann A. Glass C. (2012). The transcriptional specificity of NF-κB dimers is coded within the κB DNA response elements. Cell Rep. 2, 824–839. 10.1016/j.celrep.2012.08.042 23063365 PMC4167904

[B53] Westerheide S. D. Mayo M. W. Anest V. Hanson J. L. Baldwin A. S. JR. (2001). The putative oncoprotein Bcl-3 induces cyclin D1 to stimulate G(1) transition. Mol. Cell. Biol. 21, 8428–8436. 10.1128/MCB.21.24.8428-8436.2001 11713278 PMC100006

[B54] Wu L. Bernal G. M. Cahill K. E. Pytel P. Fitzpatrick C. A. Mashek H. (2018). BCL3 expression promotes resistance to alkylating chemotherapy in gliomas. Sci. Transl. Med. 10, eaar2238. 10.1126/scitranslmed.aar2238 29973405 PMC6613219

[B55] Yilmaz Z. B. Kofahl B. Beaudette P. Baum K. Ipenberg I. Weih F. (2014). Quantitative dissection and modeling of the NF-κB p100-p105 module reveals interdependent precursor proteolysis. Cell Rep. 9, 1756–1769. 10.1016/j.celrep.2014.11.014 25482563

[B56] Zhang X. Wang H. Claudio E. Brown K. Siebenlist U. (2007). A role for the IkappaB family member Bcl-3 in the control of central immunologic tolerance. Immunity 27, 438–452. 10.1016/j.immuni.2007.07.017 17869136 PMC2000815

[B57] Zou Y. Uddin M. M. Padmanabhan S. Zhu Y. Bu P. Vancura A. (2018). The proto-oncogene Bcl3 induces immune checkpoint PD-L1 expression, mediating proliferation of ovarian cancer cells. J. Biol. Chem. 293, 15483–15496. 10.1074/jbc.RA118.004084 30135206 PMC6177577

