# Supplementary material for: Atypical IκB Bcl3 enhances the generation of the NF-κB p52 homodimer
Source: Front Cell Dev Biol. 2022 Aug 5;10:930619. doi: 10.3389/fcell.2022.930619 (PMC9389042; doi:10.3389/fcell.2022.930619)
Supplement: Supplementary file 1 [file Presentation1.pdf]

Supplementary Information

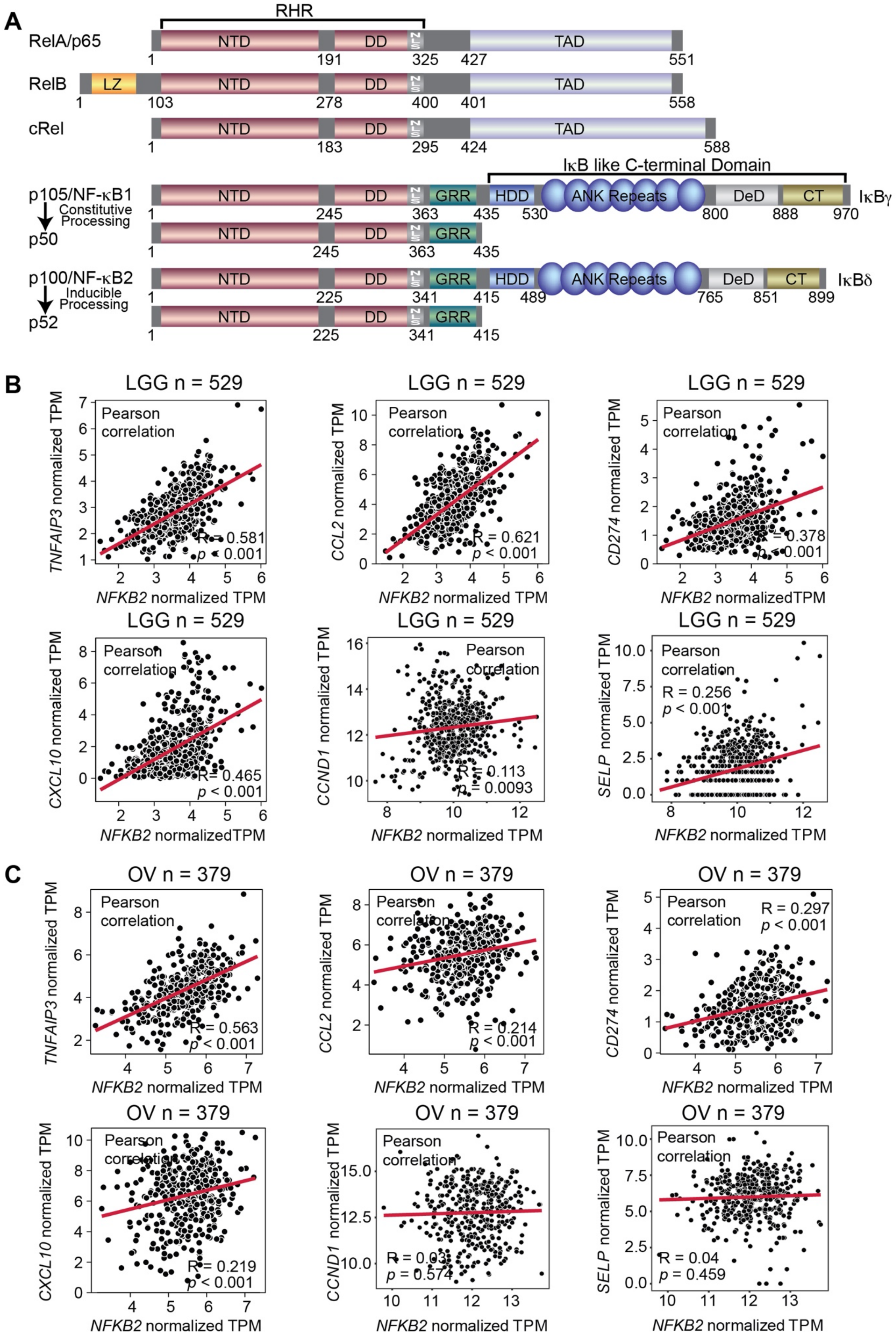

Figure S1. Correlation analysis of *NFKB2*, related to Figure 1.
(A) Domain organization of NF-κB subunits. The NF-κB family is characterized by having the rel homology region (RHR). The RHR is folded into two distinct domains, N-terminal Domain (NTD) and dimerization domain (DD). All NF-κB proteins contains a nuclear localization signal (NLS). RelA, RelB and cRel subunits (Class II) contain a transcriptional activation domain (TAD). RelB also has an N-terminal leucine zipper (LZ) domain. The p50 and p52 subunits (Class I) are generated from their precursor proteins NF-κB1/p105 and NF-κB2/p100, respectively. The precursor proteins contain an Ankyrin (ANK) repeat domain in their C-termini, function as inhibitor of NF-κB. The precursors also contain a glycine rich region (GRR), a helical dimerization domain (HDD), a death domain (DeD) and a C-terminal domain (CT).
(B-C) Scatter plots showing the correlation between mRNA expression of *NFKB2* and target genes (*TNFAIP3*, *CCL2*, *CD274*, *CXCL10*, *CCND1* and *SELP*) in (C) brain lower grade glioma (LGG) and (D) ovarian serous cystadenocarcinoma (OV) from TCGA database. “n” denotes the sample number.

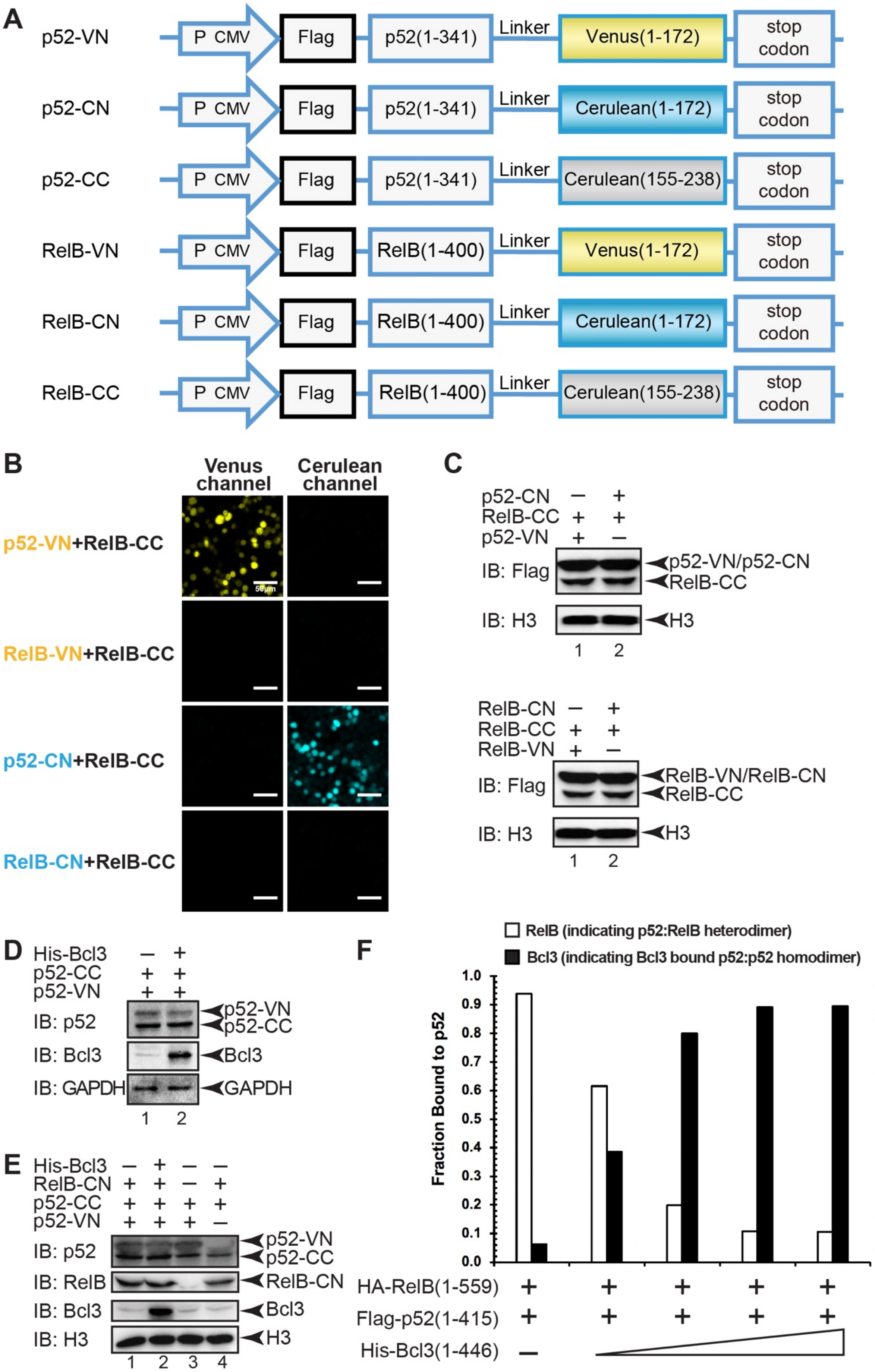
A
p52-VN
P CMV
Flag
p52(1-341)
Linker
Venus(1-172)
stop codon
p52-CN
Cerulean(1-172)
p52-CC
Cerulean(155-238)
RelB-VN
RelB(1-400)
RelB-CN
RelB-CC
B
Venus channel
Cerulean channel
p52-VN+RelB-CC
RelB-VN+RelB-CC
p52-CN+RelB-CC
RelB-CN+RelB-CC
50μm
C
IB: Flag
IB: H3
p52-VN/p52-CN
RelB-CC
H3
RelB-VN/RelB-CN
D
His-Bcl3
IB: p52
IB: Bcl3
IB: GAPDH
p52-VN
p52-CC
Bcl3
GAPDH
E
IB: RelB
RelB-CN
F
RelB (indicating p52:RelB heterodimer)
Bcl3 (indicating Bcl3 bound p52:p52 homodimer)
Fraction Bound to p52
HA-RelB(1-559)
Flag-p52(1-415)
His-Bcl3(1-446)

Figure S2. Construction of NF-κB multicolor BiFC system, related to Figure 2.
(A) Diagrams showing the p52-VN, -CN, -CC; and RelB-VN, -CN and -CC BiFC constructs.
(B) Fluorescent images with the co-expression of indicated plasmids. p52-VN+RelB-CC brings VN and CN together to form a fluorescent protein which could be detected at Venus channel; and p52-CN+RelB-CC brings CC and CN together to form an intact Cerulean protein, both fluorescent signals indicating the p52:RelB heterodimer formation in cells. However, neither RelB-VN+RelB-CC nor RelB-CN+RelB-CC generated any fluorescent signals since RelB does not form homodimer in cells.
(C) Expression of p52-VN, p52-CN, RelB-CC, RelB-VN and RelB-CN in HEK 293T cells detected by immunoblot.
(D) Expression of p52-VN and p52-CC in HEK 293T cells with and without Bcl3 detected by immunoblot.
(E) Expression of p52-VN, p52-CC and RelB-CN in HEK 293T cells with and without Bcl3 detected by immunoblot.
(F) Quantification of western blot band intensities in Figure 2F. ImageJ was used to measure the band intensity; fraction bound to p52 was calculated by the band intensity of RelB or Bcl3 divided by the total band intensities of RelB and Bcl3.

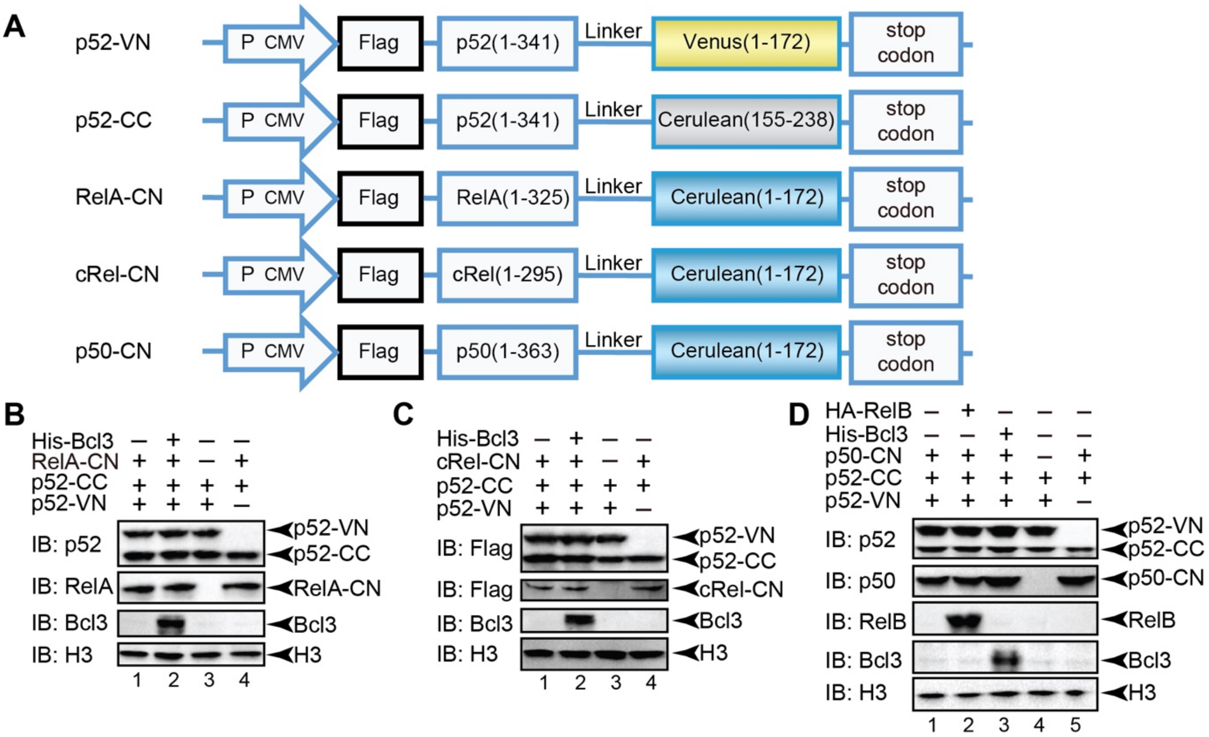

Figure S3. Expression of p52, RelA, cRel and p50 BiFC constructs, related to Figure 3. (A) Diagrams showing the p52-VN, p52-CC, RelA-CN, cRel-CN and p50-CN BiFC constructs. (B-D) Expressions of His-Bcl3, p52-VN, p52-CC and (B) RelA-CN, (C) cRel-CN, and (D) p50-CN and HA-RelB in HEK 293T cells detected by immunoblot.

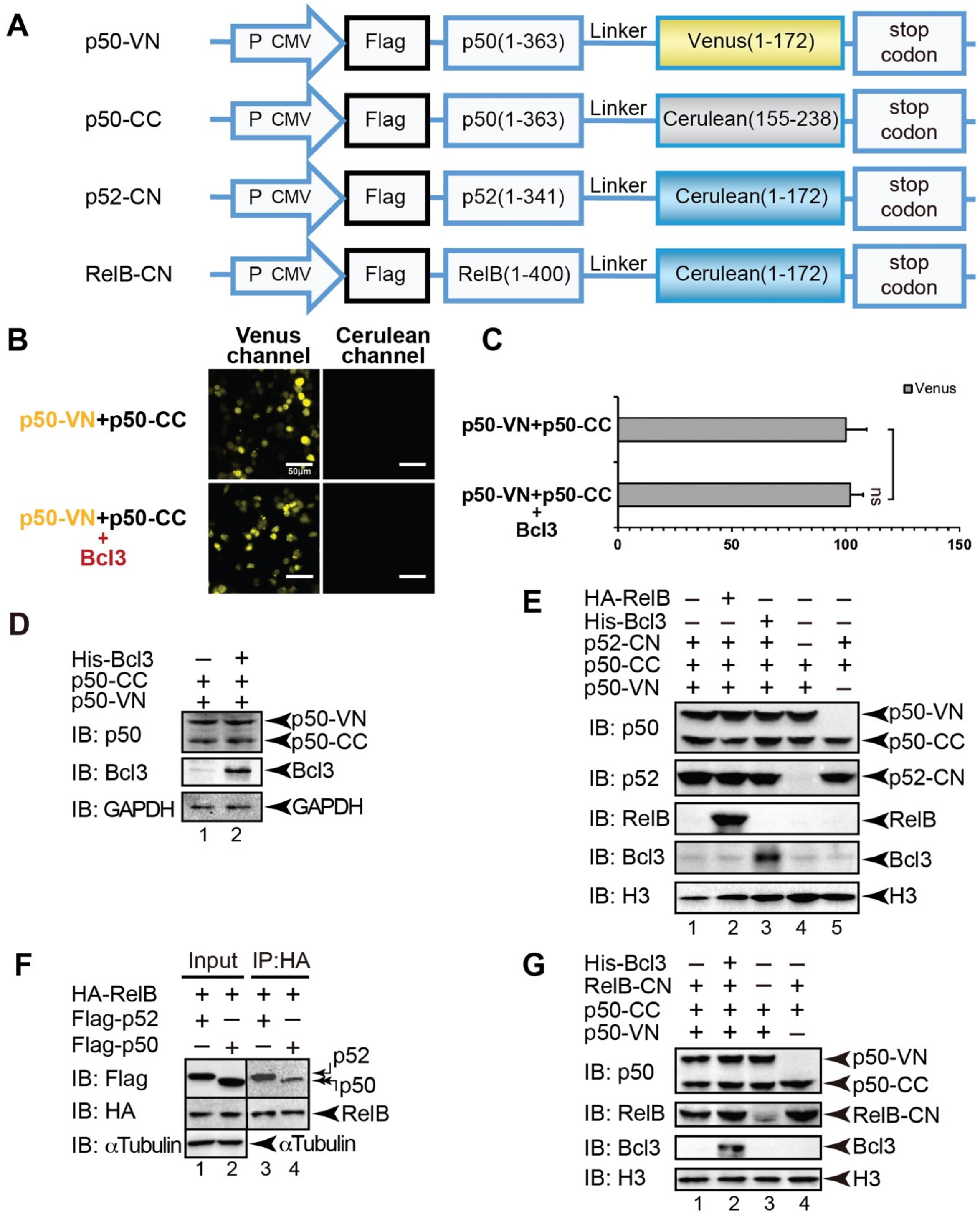

Figure S4. p50 and p52 or RelB multicolor BiFC system, related to Figure 4.
(A) Diagrams showing the p50-VN, p50-CC, p52-CN and RelB-CN BiFC constructs.
(B) Fluorescent images with the co-expression of indicated plasmids. The addition of Bcl3 did not further enhance the fluorescent intensity of p50-VN and p50-CC.
(C) Quantification of fluorescent intensity in (B); the data were analyzed from images of three independent experiments. n.s., not significant versus no Bcl3 co-expression (t test). Error bars represent SD.
(D) Expression of p52-VN and p52-CC in HEK 293T cells with and without His-Bcl3 detected by immunoblot.
(E) Expression of p50-VN, p50-CC and p52-CN in HEK 293T cells with and without HA-RelB or His-Bcl3 detected by immunoblot.

(F) Co-IP showing HA-RelB interacted with more Flag-p52 than Flag-p50.
(G) Expression of p50-VN, p50-CC and RelB-CN in HEK 293T cells with and without His-Bcl3 detected by immunoblot.

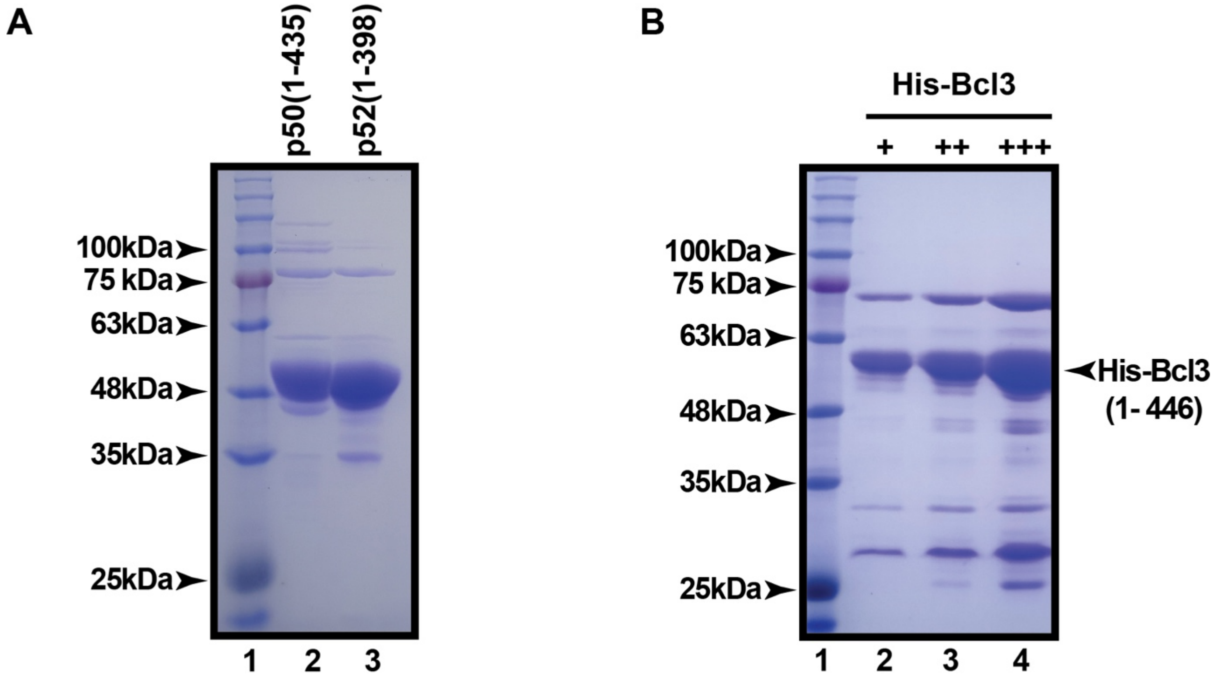

Figure S5. Recombinant Bcl3, p52 and p50 proteins, related to Figure 5.
(A-B) SDS-PAGE analysis showing the purity of (A) the non-tagged p52 and p50 proteins; (B) His-tagged Bcl3.

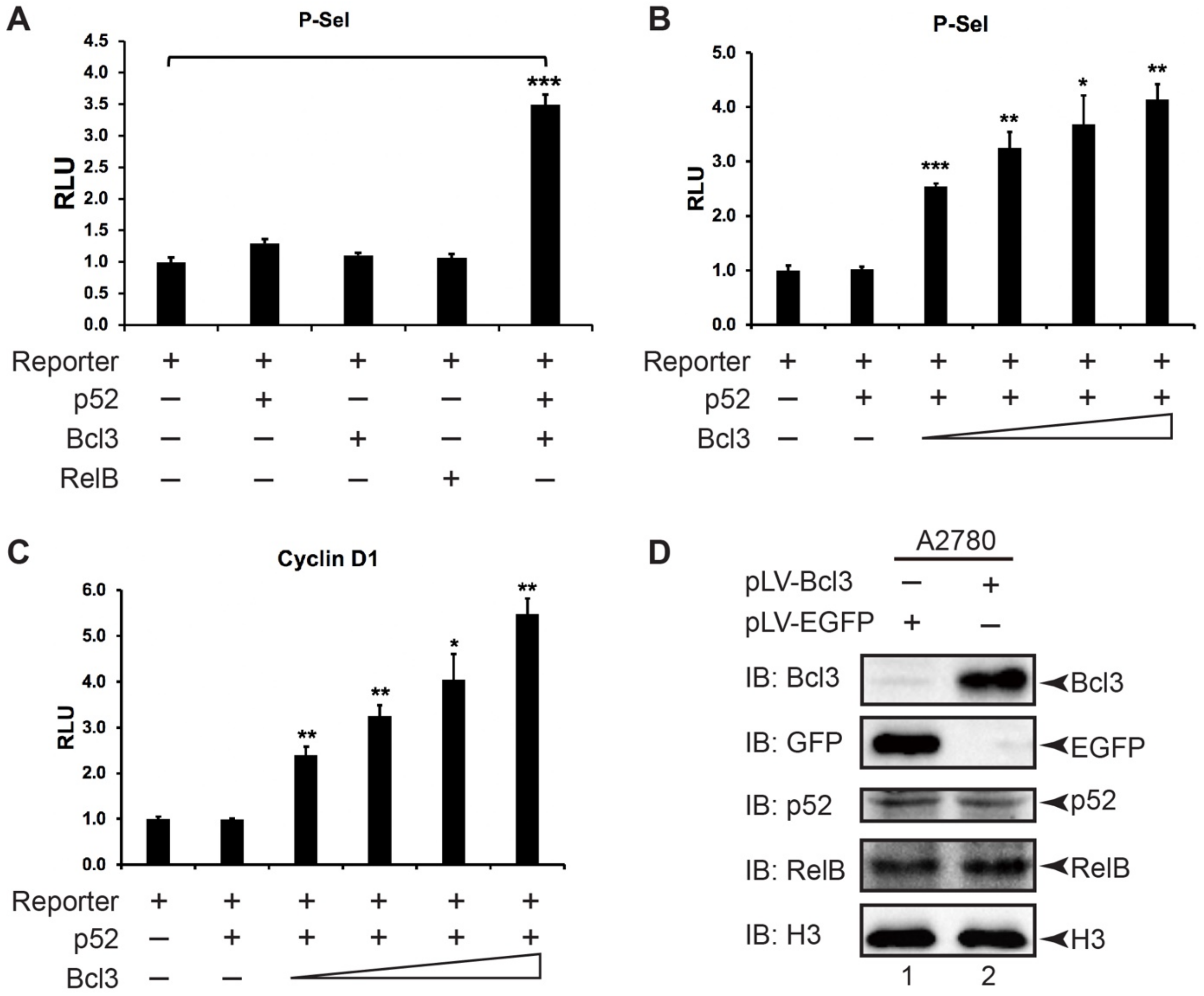

Figure S6. Bcl3 increases the expression of p52:p52 homodimer target genes, related to Figure 6.

(A) Expression of either p52, RelB or Bcl3 alone does not activate the P-Selectin luciferase reporter; co-expression of p52 and Bcl3 activates the reporter.

(B-C) P-Selectin in (B) and Cyclin D1 in (C) luciferase reporter activities were increased with increasing Bcl3 co-expression. Reporter plasmid encoding P-Selectin or Cyclin D1 κB site were co-transfected with a constant amount of p52 and an increasing amount of Bcl3 into HeLa cells. The data were analyzed from three independent experiments performed in triplicate. RLU, relative luciferase unit. *$p < 0.05$; **$p < 0.01$; ***$p<0.001$ versus reporter only control (t test). Error bars represent standard deviation (SD).

(D) Western blot analysis showing the overexpression of Bcl3 in A2780 cells by lentivirus transduction. pLV-EGFP was used as infection control. Bcl3 overexpression does not affect the endogenous level of p52 and RelB.
